# Single-Cell Transcriptome Analysis Reveals Mesenchymal Stem Cells in Cavernous Hemangioma

**DOI:** 10.3389/fcell.2022.916045

**Published:** 2022-07-05

**Authors:** Fulong Ji, Yong Liu, Jinsong Shi, Chunxiang Liu, Siqi Fu, Heng Wang, Bingbing Ren, Dong Mi, Shan Gao, Daqing Sun

**Affiliations:** ^1^ Department of Paediatric Surgery, Tianjin Medical University General Hospital, Tianjin, China; ^2^ College of Life Sciences, Nankai University, Tianjin, China; ^3^ National Clinical Research Center of Kidney Disease, Jinling Hospital, Nanjing University School of Medicine, Nanjing, China; ^4^ Department of Clinical Laboratory, Affiliated Maternity Hospital, Nankai University, Tianjin, China

**Keywords:** MSC, UCHL1, vascular tumour, stem cell, scRNA-seq

## Abstract

A cavernous hemangioma, well-known as vascular malformation, is present at birth, grows proportionately with the child, and does not undergo regression. Although a cavernous hemangioma has well-defined histopathological characteristics, its origin remains controversial. In the present study, we characterized the cellular heterogeneity of a cavernous hemangioma using single-cell RNA sequencing (scRNA-seq). The main contribution of the present study is that we discovered a large number of embryonic mesenchymal stem cells (MSCs) in a cavernous hemangioma and proposed that cavernous hemangiomas may originate from embryonic MSCs. Further analysis of the embryonic MSCs revealed that: 1) proinflammatory cytokines and related genes *TNF*, *TNFSF13B*, *TNFRSF12A*, *TNFAIP6*, and *C1QTNF6* are significantly involved in the MSC-induced immune responses in cavernous hemangiomas; 2) *UCHL1* is up-regulated in the embryonic MSC apoptosis induced by proinflammatory cytokines; 3) the UCHL1-induced apoptosis of MSCs may play an important role in the MSC-induced immune responses in cavernous hemangiomas; and 4) *UCHL1* can be used as a marker gene to detect embryonic MSCs at different apoptosis stages. In addition to MSCs, ECs, macrophages, T lymphocytes and NKCs were intensively investigated, revealing the genes and pathways featured in cavernous hemangiomas. The present study revealed the origin of cavernous hemangiomas and reported the marker genes, cell types and molecular mechanisms, which are associated with the origin, formation, progression, diagnosis and therapy of cavernous hemangiomas. The better understanding of the MSC-induced immune responses in benign tumours helps to guide future investigation and treatment of embryonic MSC-caused tumours. Our findings initiated future research for the rediscovery of MSCs, cancers/tumours and the UCHL1-induced apoptosis.

## Introduction

Vascular tumours include hemangioma, hemangioendothelioma, angiosarcoma, and their epithelioid variants (Errani et al., 2012). According to the sizes of the affected vessels, hemangiomas are histologically classified as capillary, cavernous, or mixed-type hemangiomas (Fu et al., 2020). A capillary hemangioma (superficial, red, raised), also called strawberry hemangioma, is a tumour of infancy that undergoes a phase of rapid growth and expansion followed by a period of slow but steady regression during childhood. In contrast, a cavernous hemangioma (deep dermal, blue hue), which is now classified as vascular malformation according to the International Society for the Study of Vascular Anomalies (ISSVA) classification (Wang et al., 2019), is present at birth, grows proportionately with the child, and does not undergo regression (Beck and Gosain, 2009). Cavernous hemangiomas have been reported to arise at various sites, including the skin and subcutaneous layers of the head and neck, face, extremities, liver, gastrointestinal tract, and even the thymus (Kim et al., 2018). The tumours are composed of dilated vascular spaces, with thinned smooth muscle walls separated by a variable amount of fibroconnective tissue. Major features used to discriminate cavernous hemangiomas from capillary hemangiomas include the observation of “normal” vascular endothelial cells in cavernous hemangiomas and the over-expression of vascular endothelial growth factor A (*VEGFA*) and fibroblast growth factor receptor 1 (*FGFR1*) in capillary hemangiomas during the proliferative stage (Tan et al., 2000).

Three classes of cavernous hemangiomas, hepatic cavernous hemangioma (HCH), retinal cavernous hemangioma (RCH), and cerebral cavernous hemangioma (CCH) that is also known as cerebral cavernous malformation (CCM) are comparatively well studied. As the most common benign tumour of the liver, HCH is present in up to 7% of individuals that participate in autopsy studies. Large HCHs may be associated with thrombosis, scarring, and calcification (Roberts, 2012). Histological examination of the lesions has revealed a network of vascular spaces lined by endothelial cells and separated by a thin fibrous stroma. RCH is composed of clusters of saccular aneurysms filled with dark blood. Microscopic examination of the lesions has revealed multiple thin-walled interconnected vascular spaces lined by flat endothelial cells, with red cell necrosis and partially organised intravascular thrombosis. Furthermore, the vascular spaces in RCHs are bordered by thin, fibrous septa, with occasional nerve fibers and glial cells. CCHs that occur in the central nervous system, most often in the brain, can cause intracranial hemorrhage, seizures, neurological deficits, and even death. CCH has sporadic and familial forms; familial CCHs often display multiple lesions and autosomal dominant inheritance. Ultrastructural studies revealed abnormal or absent blood–brain barrier components, poorly formed tight junctions with gaps between endothelial cells, lack of astrocytic foot processes, and few pericytes in CCHs. Although mutations in the *KRIT1* and *CCM1* genes have been found in patients with both RCH and CCH, the cause of RCH is still unknown (Wang and Chen, 2017). One possible cause of familial CCHs is the loss-of-function mutations in three genes, *KRIT1*, *CCM2*, and *PDCD10* (Kim et al., 2016). According to the current theory, hemangiomas originate from neogenesis or revival of dormant embryonic angioblasts and arise through hormonally driven vessel growth (Beck and Gosain, 2009). However, the origins of hemangiomas remain controversial.

Although capillary hemangiomas are mainly treated by surgery, several drugs (*e.g.,* propranolol and glucocorticoids) have been developed to avoid the risks of intraoperative profuse bleeding, postoperative recurrence, long-term scarring, and other complications. The treatment of cavernous hemangiomas is still dependent on excision and venous embolization therapy (Wang et al., 2019). However, inadequate excision causes recurrence (Jang et al., 2011). To develop drugs or other new therapies, more basic research must be conducted to better understand cavernous hemangiomas at the molecular level. In the present study, we characterised the cellular heterogeneity of a cavernous hemangioma using single-cell RNA sequencing (scRNA-seq) (Gao, 2018). Through further analysis of the scRNA-seq data, we aimed to: 1) reveal the comprehensive cellular composition and gene expression profile of a cavernous hemangioma at the single-cell level, and 2) discover the marker genes, cell types, and molecular mechanisms, which are associated with the origin, formation, progression, diagnosis, and therapy of cavernous hemangiomas.

## Results

### Single-Cell RNA Sequencing and Basic Analyses

**FIGURE 1:**
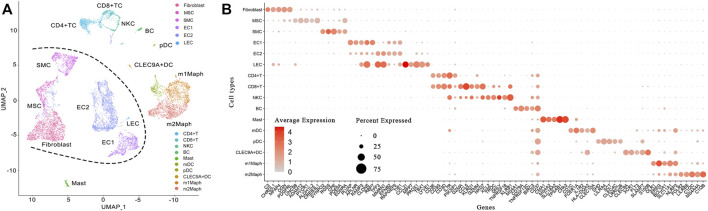
Identification of 16 cell types in a cavernous hemangioma. A total of 10,784 cells were finally clustered into 16 clusters and then identified as 16 cell types. The non-immune cell types (fibroblast, MSC, SMC, EC1, EC2 and LEC) were clearly separated from immune cell types (CD4+TC, CD8+TC, NKC, BC, mast, mDC, pDC, CLEC9A + DC, m1Maph and m2Maph) by a borderline (named Nankai borderline in the present study). **(A)**. Uniform Manifold Approximation and Projection (UMAP) method was used to show the clustering results; **(B)**. For each of the 16 clusters, a combination of five marker genes (the symbols of 80 genes can be seen in Table 1) were assigned. MSC: mesenchymal stem cell, SMC: smooth muscle cell, EC1: endothelial cell type 1, EC2: endothelial cell type 2, LEC: lymphatic endothelial cell, CD4+TC: CD4 positive T cell, CD8+TC: CD8 positive T cell, NKC: natural killer cell, BC: B cell, mDC: monocyte derived dendritic cell, pDC: plasmacytoid dendritic cell, CLEC9A + DC: CLEC9A positive dendritic cell, m1Maph: M1-like macrophage and m2Maph: M2-like macrophage.

The lesion was obtained from a 6-year-old patient diagnosed with a cavernous hemangioma (Supplementary File S1). Using a piece of tissue in the centre of the tumour, scRNA-seq libraries (10x Genomics, United States) were constructed and sequenced to produce ∼163 Gbp of raw data (Materials and Methods). After data cleaning and quality control, a total of 10,784 cells and 22,023 genes were identified to produce a 22,010 × 10,784 matrix and a 13 × 10,784 matrix, representing the expression levels of nuclear and mitochondrial genes, respectively. Using the 22,010 × 10,784 nuclear matrix, 10,784 cells were clustered into 18 clusters with adjusted parameters (Materials and Methods). The identification of each cluster as a specific cell type took two-steps (the rough and exact identification). Firstly, 18 clusters were roughly identified as 18 cell types. Next, three types of endothelial cells were merged into one type. Then, 18 clusters were merged into 16 clusters, which were named according to their cell types (Figure 1A), including fibroblast cell type 1 (fibroblast1), type 2 (fibroblast2), smooth muscle cell (SMC), endothelial cell type 1 (EC1), type 2 (EC2), lymphatic endothelial cell (LEC), T lymphocyte type 1 (TC1), type 2 (TC2), type 3 (TC3), B lymphocyte (BC), mast cell, monocyte derived dendritic cell (mDC), plasmacytoid dendritic cell (pDC), CLEC9A positive dendritic cell (CLEC9A + DC), macrophage type 1 and type 2, respectively. For each of the 16 clusters, differential expression analysis between cells inside and outside the cluster was performed to produce a gene-expression signature (Materials and Methods) including all differentially expressed (DE) genes (Supplementary File S2). By further analysis of gene-expression signatures, 16 clusters were exactly identified as 16 cell types and then renamed as fibroblast, mesenchymal stem cell (MSC), SMC, EC1, EC2, LEC, CD4 positive T cell (CD4+TC), CD8 positive T cell (CD8+TC), natural killer cell (NKC), BC, mast, mDC, pDC, CLEC9A + DC, M1-like macrophage (m1Maph) and M2-like macrophage (m2Maph) clusters, respectively. To confirm the presence of these cell types in the tumour, dilated capillaries, normal capillaries, lymphatic vessels, muscle tissue, and connective tissue were observed by haematoxylin–eosin (HE) staining (Supplementary File S1). As no single gene can be used as a marker to discriminate a cell type from its relatives (*e.g.*, CD8+T from CD4+T), we assigned the top five DE genes as a combination of marker genes (Materials and Methods) to each cell type to help to identify these cell types in future studies (Table 1). The representation of each cell type by the combination of marker genes was also displayed in Venn diagrams (Supplementary Figure S4). We designed a new metric (Materials and Methods), named the union and intersection coverage of a cluster (UICC), to evaluate the representation of a cell type by a combination of marker genes.

**TABLE 1:** Marker genes of 16 cell types.

Cell Type	Cell#	Marker Genes	UICC
Fibroblast	2033	C3, CHRDL1, MFAP4, OGN, PDGFRL	0.27
MSC	610	UNC5B, ADAM12, PYCR1, ALDH1L2, CREB3L1	0.31
SMC	881	SYNPO2, RGS5, CPE, PDE5A, EDNRA	0.26
EC1	1,032	PLVAP, APLNR, RAMP3, CLDN5, RBP7	0.29
EC2	2,273	TFF3, MMRN1, EFNB2, RAPGEF5, TIE1	0.26
LEC	38	CCL21, MPP7, PROX1, TBX1, LYVE1	0.37
CD4+TC	596	CD3E, CD3G, CD3D, IL7R, PIK3IP1	0.22
CD8+TC	307	GZMA, CCL5, KLRD1, GZMM, NKG7	0.29
NKC	201	XCL2, TRDC, GNLY, TNFRSF18, KLRB1	0.27
BC	119	MS4A1, CD79A, TNFRSF13C, BIRC3, CD37	0.25
Mast	137	CMA1, MS4A2, SLC18A2, TPSAB1, CPA3	0.47
mDC	266	CD1C, FCER1A, IL1R2, HLA-DQA2, CLEC10A	0.28
pDC	36	SHD, LILRA4, SCT, CLEC4C, LRRC26	0.14
CLEC9A + DC	47	CLEC9A, XCR1, FLT3, SLAMF7, S100B	0.30
m1Maph	922	OLR1, EREG, BCL2A1, SLC11A1, NLRP3	0.32
m2Maph	1,286	FOLR2, LILRB5, C1QC, MS4A4A, C1QB	0.46

A total of 10,784 cells were clustered into 16 clusters and then identified as 16 cell types (**the first column**) with different numbers (**the second column**). For each of the 16 clusters, a combination of five marker genes were assigned (**the third column**). The differential expression analysis between the cells inside and outside the cluster was performed to select the top five DE, genes as a combination of five marker genes. UICC (union and intersection coverage of a cluster) is calculated (**the fourth column**) by multiplying UCC (union coverage of a cluster) by ICC (intersection coverage of a cluster). MSC: mesenchymal stem cell, SMC: smooth muscle cell, EC1: endothelial cell type 1, EC2: endothelial cell type 2, LEC: lymphatic endothelial cell, CD4+TC: CD4 positive T cell, CD8+TC: CD8 positive T cell, NKC: natural killer cell, BC: B cell, mDC: monocyte derived dendritic cell, pDC: plasmacytoid dendritic cell, CLEC9A + DC: CLEC9A positive dendritic cell, m1Maph: M1-like macrophage and m2Maph: M2-like macrophage.

In a previous study of ascending thoracic aortic aneurysms (ATAAs) (Li et al., 2020), 11 major cell types were identified by known marker genes and they were fibroblast, MSC, SMC1, SMC2, EC, TC, NKC, BC, mast cell, MonoMaphDC (monocyte/macrophage/DC) and plasma cell. Thus, we performed a simple comparison between cell populations in the cavernous hemangioma tissue and those in ATAA tissues and their controls (Li et al., 2020) and found several main differences, particularly, the cavernous hemangioma tissue contained: 1) lower proportion of immune cells (36.32% of the total cells) than ATAA tissues (64.08%), and more than their controls (26.24%); 2) higher proportions of fibroblasts and ECs (24.51 and 30.65%) than ATAA tissues (7.6 and 7.43%) and their controls (13.51 and 14.02%); 3) similar proportion of DCs and macrophages (21.73%) to ATAA tissues (23.71%), much more than the controls of ATAA (7.64%); and 4) much lower proportions of B and plasma cells (1.27% and 0) than ATAA tissues (9.36 and 11.5%) and their controls (4.31 and 2.09%). The above results signified that fibroblasts, ECs, and macrophages in the cavernous hemangioma tissue need to be intensively investigated.

### Identification of Fibroblasts and Mesenchymal Stem Cells

The fibroblast1 and fibroblast2 clusters contained 18.85% (2,033/10,784) and 5.66% (610/10,784) of the total cells, respectively. Such proportion of fibroblast1 (18.85%) was markedly higher than that of SMCs (8.17%, 881/10784), which was consistent with the prominent histological features of cavernous hemangiomas where the thinned smooth muscle walls are separated by a variable amount of fibroconnective tissue (Introduction). Fibroblast1 was finally identified as a cluster of fibroblasts, as it highly expressed all the known marker genes, *PDGFRA*, *PDGFRB*, *MEG3*, *SCARA5*, *COL14A1*, and *OGN* (Supplementary Figure S5), which have been reported in a previous study (Kuppe et al., 2021). In contrast, fibroblast2 expressed *PDGFRB* and *MEG3* at high levels (0.5 < LFCio ≤ μ+σ), *PDGFRA* and *COL14A1* at similar levels (|LFCio-μ|≤0.5), *OGN* at a low level (μ-σ<LFCio<=-0.5), and *SCARA5* at a very low level (μ-2σ<LFCio<=-μ-σ), compared with other cells. Although both fibroblast1 and fibroblast2 expressed a number of fibrillin, fibulin, collagen, and elastin genes required in the extracellular matrix (ECM), including *FBN1*, *FBLN1*, *FBLN2*, *FBLN5*, *COL1A1*, *COL1A2*, *COL3A1*, *COL6A1*, *COL6A2*, *COL5A2*, *COL14A1*, and *ELN* (Supplementary Figure S6), fibroblast1 markedly higher expressed most of these genes (*e.g.*, *FBLN1*, *FBLN2*, and *ELN*) than fibroblast2. According to the previous study (Kuppe et al., 2021), pericytes, closely related to fibroblasts, also highly express ECM genes. Then, we examined the expression levels of *PDGFRA*, *NOTCH3*, and *RGS5* that are three marker genes of pericytes (Li et al., 2020) (Supplementary Figure S5). Pericytes expressed *PDGFRA* at a low level, while fibroblast2 expressed *PDGFRA* at a similar level, compared to other cells. Although both fibroblast2 and pericytes expressed *NOTCH3* at high levels, fibroblast2 did not originate from pericytes, as pericytes expressed *RGS5* at a high level, but fibroblast2 expressed *RGS5* at a very low level, compared to other cells. The results in this paragraph indicated that fibroblast2 is a cluster of cells related to, but markedly different from fibroblasts.

**FIGURE 2:**
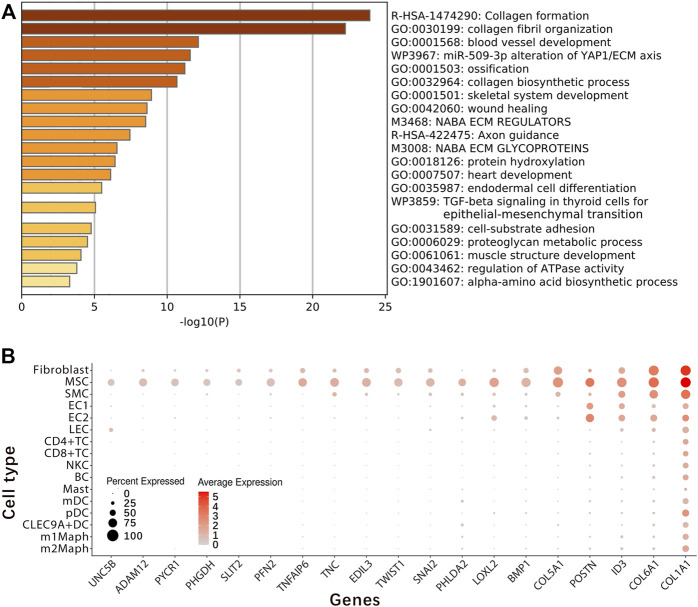
Identification of embryonic mesenchymal stem cells. Differential expression analysis between cells inside and outside fibroblast2 was performed to generate a gene-expression signature (Supplementary File S2). From the gene-expression signature, the 63 coding genes and a long noncoding RNA (lncRNA) gene *RP11-14N7.2* (Table 2) were selected for the exact identification of fibroblast2. Finally, we concluded that fibroblast2 is a cluster of embryonic mesenchymal stem cells (MSCs). **(A)**. GO and pathway annotation of the 63 coding genes with analysis were performed using the Metascape website; **(B)**. At least 19 genes (*UNC5B*, *ADAM12*, *PYCR1*, *PHGDH*, *SLIT2*, *PFN2*, *TNFAIP6*, *TNC*, *EDIL3*, *TWIST1*, *SNAI2*, *PHLDA2*, *LOXL2*, *BMP1*, *COL5A1*, *POSTN*, *ID3*, *COL6A1*, and *COL1A1*) have been reported to be over-expressed or up-regulated in stem cells, and among these 19 genes, seven (*PYCR1*, *TNFAIP6*, *EDIL3*, *TWIST1*, *LOXL2*, *BMP1*, and *COL1A1*) have been reported to be expressed in MSCs in the previous studies (Supplementary File S3).

For exact identification of fibroblast2, differential expression analysis between cells inside and outside fibroblast2 was performed to generate a gene-expression signature (Supplementary File S2). From the gene-expression signature, 63 coding genes and a long noncoding RNA (lncRNA) gene *RP11-14N7.2* (Table 2) were selected for further analysis. Gene ontology (GO) and pathway annotation of the 63 coding genes (Figure 2A) revealed that 57.14% (36/63) of the genes are involved in ECM organisation (GO:0030198), and 15.87% (10/63) of the genes are involved in the response to fibroblast growth (GO:0071774). This finding confirmed that fibroblast2 is a cluster of cells related to fibroblasts. Significantly, many GO annotations were found to be enriched in endodermal cell differentiation and tissue development, including endodermal cell differentiation (GO:0035987), blood vessel development (GO:0001568), skeletal system development (GO:0001501), heart development (GO:0007507), bone development (GO:0060348), muscle organ development (GO:0007517), reproductive structure development (GO:0048608), and lung development (GO:0030324). In particular, *TWIST1*, *SULF1*, *COL5A1*, *COL5A2*, *COL6A1*, and *FN1* are associated with endodermal cell differentiation (GO:0035987), thus indicating the characteristics of stem or progenitor cells in fibroblast2. Searching literature databases using the 63 coding genes revealed that at least 19 genes had been reported to be over-expressed or up-regulated in stem cells, namely, *UNC5B*, *ADAM12*, *PYCR1*, *PHGDH*, *SLIT2*, *PFN2*, *TNFAIP6*, *TNC*, *EDIL3*, *TWIST1*, *SNAI2*, *PHLDA2*, *LOXL2*, *BMP1*, *COL5A1*, *POSTN*, *ID3*, *COL6A1*, and *COL1A1* (Figure 2B). Among the 19 genes, seven (*PYCR1*, *TNFAIP6*, *EDIL3*, *TWIST1*, *LOXL2*, *BMP1,* and *COL1A1*) have been reported to be expressed in MSCs in the previous studies (Supplementary File S3). Among these seven genes, *TWIST1* is a basic helix-loop-helix (bHLH) transcription factor that plays essential and pivotal roles in multiple stages of embryonic development. The over-expression of *TWIST1* induces epithelial-mesenchymal transition (EMT), a key process in cancer metastasis (Zhu et al., 2016). Among the 19 genes, *UNC5B*, *ADAM12*, and *PYCR1* belong to the five marker genes of fibroblast2 (Table 1). Notably, *UNC5B* has been reported to be a marker gene of epiblast stem cells. According to annotations from the GeneCards database (Marilyn et al., 2002), *UNC5B* encodes a member of the netrin family of receptors, and the encoded protein mediates the repulsive effect of netrin-1. The protein encoded by *UNC5B* belongs to a group of proteins called dependence receptors (DpRs), which are involved in embryogenesis and cancer progression. The results in this paragraph indicated that fibroblast2 is a cluster of MSCs.

**TABLE 2:** The gene-expression signature of mesenchymal stem cells.

Gene Symbol	Avg_in	LFCio	PCTin (%)	PCTout (%)	PCTin/PCTout
UNC5B	0.60	3.86	63.30	2.70	23.44
ADAM12	1.15	2.55	75.60	6.00	12.60
PYCR1	0.75	2.98	71.80	5.80	12.38
ALDH1L2	0.44	2.59	60.20	5.00	12.04
CREB3L1	1.10	3.33	79.00	6.70	11.79
UCHL1	1.22	3.33	65.90	6.30	10.46
PHGDH	0.60	2.69	66.10	6.40	10.33
GPC1	1.02	2.88	78.70	9.10	8.65
SCARF2	1.17	2.68	84.90	10.00	8.49
ENPP1	1.66	3.14	69.20	8.30	8.34
NCS1	0.56	2.43	64.10	7.70	8.32
COL5A3	3.42	3.40	94.60	11.90	7.95
SLIT2	0.59	2.14	62.80	8.10	7.75
PFN2	0.91	2.54	77.70	10.10	7.69
TNFAIP6	2.99	3.28	81.80	11.20	7.30
DCBLD2	2.35	3.63	88.70	12.70	6.98
TNC	2.55	2.32	84.60	12.20	6.93
EDIL3	2.07	2.42	84.90	12.50	6.79
RP11-14N7.2	0.82	2.22	73.80	10.90	6.77
CHPF	1.20	2.00	83.40	12.40	6.73
P4HA2	2.02	3.02	84.40	12.60	6.70
LOXL1	1.66	2.26	81.60	12.30	6.63
TWIST1	1.20	2.19	80.30	12.20	6.58
SNAI2	1.60	2.59	81.50	12.40	6.57
EMILIN1	1.78	2.56	90.00	14.30	6.29
TNFRSF12A	2.79	3.41	70.20	11.30	6.21
CCDC102B	0.93	2.01	65.70	10.80	6.08
PHLDA2	2.07	2.91	72.00	12.20	5.90
C11orf24	0.86	2.27	75.10	12.90	5.82
KDELR3	1.84	2.43	87.70	15.30	5.73
PAPSS2	0.99	2.09	78.00	14.10	5.53
CYGB	1.62	2.22	69.80	13.10	5.33
LOX	3.12	2.41	89.00	16.80	5.30
CERCAM	2.25	2.09	93.10	18.00	5.17
C12orf75	3.30	3.02	81.80	16.90	4.84
CLEC11A	3.15	2.47	96.60	20.50	4.71
PLOD2	1.55	2.16	80.20	17.70	4.53
UGDH	1.20	2.16	78.50	17.40	4.51
TUBB2A	1.47	2.08	75.20	17.80	4.22
LOXL2	4.27	2.78	91.50	22.10	4.14
BMP1	1.91	2.21	89.00	21.50	4.14
GPX8	2.10	2.01	91.60	23.00	3.98
RCN3	5.04	2.97	96.10	24.20	3.97
SULF1	2.78	2.17	85.90	22.60	3.80
COL5A1	8.90	2.50	99.30	27.60	3.60
FKBP10	2.75	2.20	95.20	26.90	3.54
TPM2	10.25	2.41	98.70	28.20	3.50
LRRC59	2.04	2.14	84.40	24.90	3.39
CKAP4	4.19	2.48	94.90	29.90	3.17
UACA	3.83	2.22	91.60	31.50	2.91
COL5A2	13.55	2.36	99.70	35.40	2.82
SERPINH1	5.16	2.12	98.00	37.30	2.63
COL6A3	39.17	2.61	99.30	39.30	2.53
PRDX4	4.79	2.08	96.40	40.10	2.40
POSTN	23.30	2.29	86.20	36.80	2.34
TPM1	8.72	2.13	97.20	42.20	2.30
ID3	9.07	2.04	93.30	43.60	2.14
CALU	7.22	2.03	98.70	49.20	2.01
COL6A1	31.81	2.41	99.80	51.60	1.93
MYDGF	6.20	2.07	96.10	50.10	1.92
FN1	52.12	2.27	99.80	67.00	1.49
COL3A1	133.81	2.02	100.00	68.30	1.46
COL1A1	210.95	2.64	100.00	69.00	1.45
COL1A2	128.04	2.07	100.00	74.10	1.35

The differential expression analysis between the cells inside and outside mesenchymal stem cells (MSCs) was performed to generate a gene-expression signature (Supplementary File S2). From the gene-expression signature, 63 coding genes and a long noncoding RNA (lncRNA) gene RP11-14N7.2 (**the first column**) were selected for further analysis, using LFCio, above 2 (**the second column**). The column 4 to 7 are PCTin (the percentage of cells that expressed a gene inside the cluster), PCTout (the percentage of cells that expressed a gene outside the cluster), LFCio [the 2-based log-transformed fold changes between the average (arithmetic mean) expression level of a gene inside and that outside a cluster], and Avg_in (the average expression level of a gene inside the cluster). LFCio, is the “log2FoldChange” calculated by DESeq2.

The identified MSCs exhibited characteristics of cancers/tumours. Notably, at least 33 genes in the gene-expression signature (Table 2) are currently being studied in cancers/tumour biology and they are: 1) seven genes (*CREB3L1, SLIT2, CHPF, TNFRSF12A, SULF1, UACA* and *TPM1*) which have been reported to be under-expressed or down-regulated (Supplementary Figure S7); 2) 25 genes (*UNC5B, ADAM12, PYCR1, PHGDH, GPC1, ENPP1, NCS1, PFN2, TNFAIP6, DCBLD2, EDIL3, P4HA2, TWIST1, SNAI2, KDELR3, CERCAM, CLEC11A, PLOD2, UGDH, LOXL2, GPX8, SERPINH1, PRDX4, POSTN* and *MYDGF*) which have been reported to be over-expressed or up-regulated (Supplementary Figure S8) and 3) *UCHL1* which has been reported as a tumour promoter in pancreatic, prostate, and lung cancers but a tumour suppressor in ovarian cancer, hepatocellular cancer, and nasopharyngeal carcinoma (Supplementary File S3). Among these 33 genes, *POSTN* encodes a secreted extracellular matrix protein that functions in tissue development and regeneration, including wound healing and ventricular remodelling following myocardial infarction. According to a previous study (Malanchi et al., 2012), *POSTN* is expressed by fibroblasts in normal tissue and the stroma of the primary tumour and plays a role in cancer stem cell maintenance and metastasis. In humans, high expression of *POSTN* has been detected in various types of cancer, including breast, ovarian, lung, prostate, kidney, intestine, and pancreas (Borecka et al., 2020). In another previous study (Kikuchi et al., 2014), the results have demonstrated the over-expression of *POSTN* in cancer-associated fibroblasts (CAFs) and suggested that *POSTN* constitutes the primary tumour niche by supporting cancer cell proliferation through the ERK signalling pathway in gastric cancer. In the present study, we found that *POSTN* was expressed in MSC, EC2, EC1, and fibroblasts clusters at levels from highest to lowest, which suggested that *POSTN* is involved in MSC differentiation. The MSCs also exhibited other characteristics of cancers/tumours, *e.g.,* high expression of *GAPDH* and very high expression of *MKI67*. *MKI67*, a tumour proliferation marker, encodes Ki-67, which is associated with cellular proliferation (Figure 4). Accordingly, the expression of *MKI67* correlates with tumour grade in many cancers. Then, we proposed that the embryonic MSCs caused the cavernous hemangioma, which need be verified by experiments in the future.

### Confirmation and Characterization of the Identified MSCs

In previous studies, MSCs have been discovered from different sources (*e.g.,* placentas, bone marrows and tumours), however, the identification of them is still difficult, as many of the discovered MSCs can not be isolated or cultured. Currently, we only have criteria from International Society for Cellular Therapy (ISCT) to roughly identify MSCs (Dominici et al., 2006), they are: 1) MSCs must be plastic-adherent when maintained in standard culture conditions using tissue culture flasks; 2) most of MSCs (more than 95%) must express *NT5E* (*CD73*), *THY1* (*CD90*) and *ENG* (*CD105*), and lack expression (only less than 2% positive) of *PTPRC* (*CD45*), *CD34*, *CD14* or *ITGAM* (*CD11b*), *CD79A* or *CD19* and HLA class II; and 3) MSCs must be able to differentiate to osteoblasts, adipocytes and chondroblasts under standard *in vitro* differentiating conditions. In general, the identified MSCs in cavernous hemangioma meet the above criteria, as they highly expressed *NT5E* and *THY1*, however, lowly or very lowly expressed *ENG*, *PTPRC*, *CD34*, *CD14*, *ITGAM*, *CD79A*, and *CD19*, compared with other cells. Eventually, the MSCs in the cavernous hemangioma were identified as embryonic MSCs, ruling out other possibilities: 1) they did not originate from pericytes (As described above); 2) they were not CAFs, as *VEGFC* and *HGF* were expressed at high levels, *TGFB1* and *TGFB3* at very high levels, *VEGFB* at an average level, *VEGFA*, *TGFB2*, *IL6*, and *CXCL12* at low levels, and *GAS6* at a very low level in the MSCs, whereas these 10 marker genes are highly expressed in CAFs (Sahai et al., 2020); 3) they were not bone marrow-derived MSCs from wound healing process, as the patient carried this tumour at birth without history of injury (Supplementary File S1); 4) they were not generated via EMT (As described above), as the tumour tissue did not contain epithelial cells, although the very high expression of *TWIST1* was detected in the MSCs; and 5) they were not transformed from angioblasts, as we only detected the low-level expression of *FGF2*, which has an essential role in induction of angioblasts with potential to transform into MSCs or hematopoietic cells (hemangioblasts) as reported in a previous study (Slukvin and Vodyanik, 2011). The above results did not rule out the probability that the MSCs in the cavernous hemangioma were generated via endothelial-mesenchymal transition (EndMT), which is crucial during embryonic development and a contributing source of CAFs that participate in tumour growth and metastasis (Mai et al., 2013). Then, we addressed a novel scientific question: do CAFs in certain cancers/tumours originate from embryonic MSCs as CAFs in glioma, breast, gastric and pancreatic cancers originate from bone marrow-derived MSCs? (Liu et al., 2019).

**FIGURE 3:**
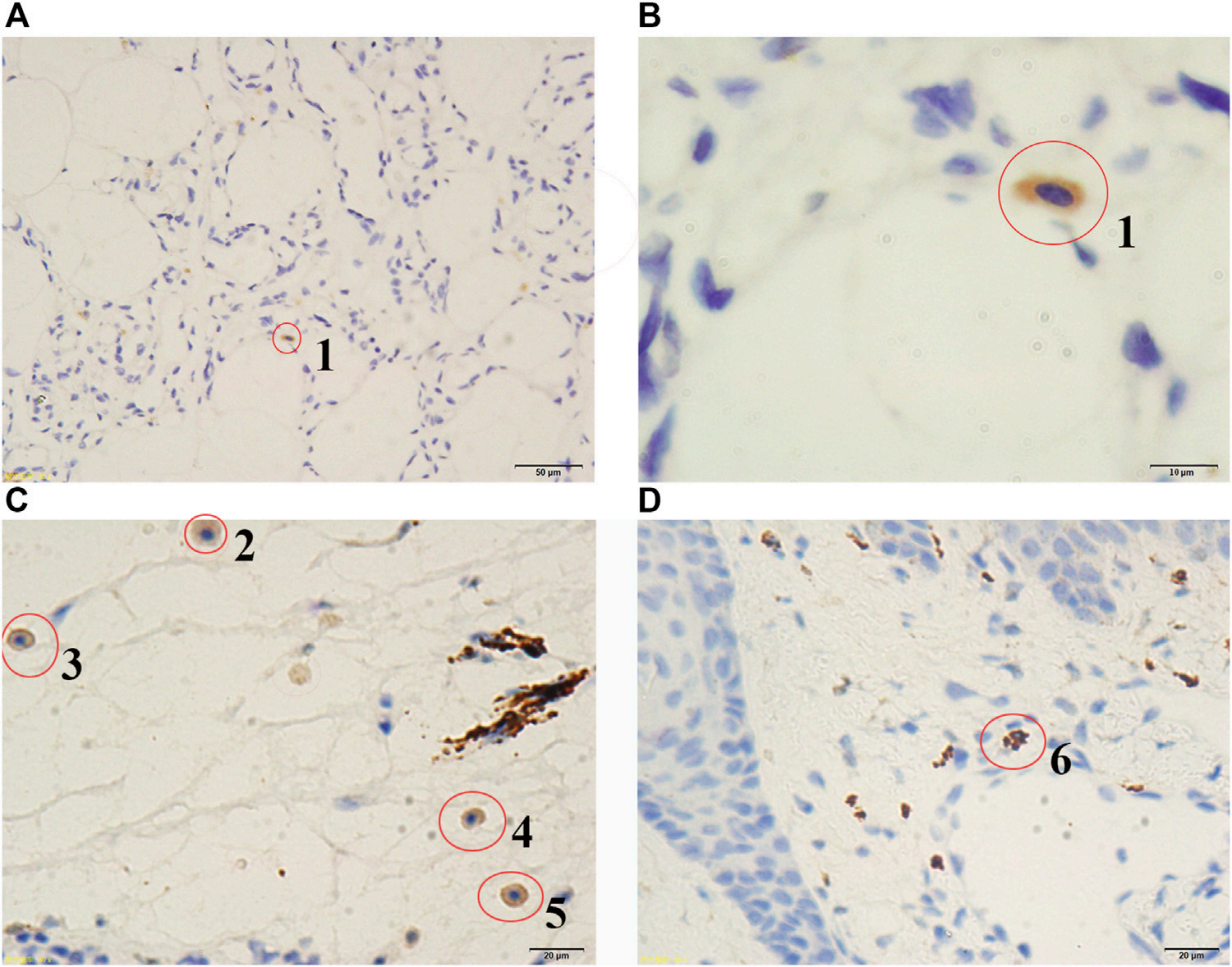
Stained embryonic MSCs at different apoptosis stages. Red circles were used to indicate the cells stained by immunohistochemistry (IHC) technique. According to the nuclear sizes, the stained cells were numbered from 1 to 6, which are embryonic mesenchymal stem cells (MSCs) at different apoptosis stages. The larger the stained nuclear size is, the earlier the apoptosis stage is. Microscopes with ordinary lens **(A)** and oil immersion lens **(B)** were used to observe tumour tissue of the patient (Admission number: 1097108). Microscopes with ordinary lens **(C and D)** were used to observe tumour tissues of the patients (Admission numbers: 1089641 and 1082186). The patient information (Admission number: 1097108, 1089641 and 1082186) can be seen in the Supplementary File S1.

Although MSCs have been reported in human solid tumours (*e.g.,* bone sarcomas, lipomas, or infantile hemangiomas), less is known about them (McLean et al., 2011) and their identities are still uncertain without a gene-expression signature of MSCs as a reference. In the present study, we discovered a large number of embryonic MSCs in a cavernous hemangioma and provided the gene-expression signature of embryonic MSCs using scRNA-seq data. This gene-expression signature can be used as a reference for the exact identification of embryonic MSCs in future studies. Among the 63 coding genes (Table 2) in the gene-expression signature, *UCHL1* was identified as the best one for discriminating embryonic MSCs from other cells. Using the antibody of UCHL1, we designed a simple and fast method to identify embryonic MSCs, based on immunohistochemistry (IHC) technique. Then, we detected embryonic MSCs in the tumour tissues of three other patients (Supplementary File S1) and the stained MSCs can be characterized by three features (Figure 3):1) the cytoplasm, not the nuclei (in oval shape), is stained by IHC; 2) the ratio of nuclear size to cell size is slightly smaller than that of an embryonic stem cell; and 3) the stained cells are often located in the connective tissue. As an unexpected and important result, most of these stained MSCs were undergoing cell apoptosis (Figure 3). The above results demonstrated that the embryonic MSCs can be detected by the antibody of UCHL1 using IHC. Although the functions of *UCHL1* remain illusive (Described as above). we still characterized it by the following experimental results from previous studies: 1) *UCHL1* induces G0/G1 cell cycle arrest and apoptosis through stabilizing p53; 2) UCHL1 concentrations in the blood plasma of boys with cryptorchidism, may reflect the heat-induced apoptosis of germ cells (Dorota et al., 2018); 3) the elevation in UCHL1 concentration is consistent with the severity of neural apoptosis following deep hypothermic circulatory arrest (DHCA) (Zhang et al., 2015) and 4) Inhibition of UCHL1 expression suppresses MSC apoptosis that is induced by proinflammatory cytokines (*i.e.* IFN-γ and TNF-α) via up-regulation of *BCL2* (Gu et al., 2018). These results suggested that the UCHL1-induced apoptosis of MSCs may play an important role in the MSC-induced immune responses in cavernous hemangiomas.

**FIGURE 4:**
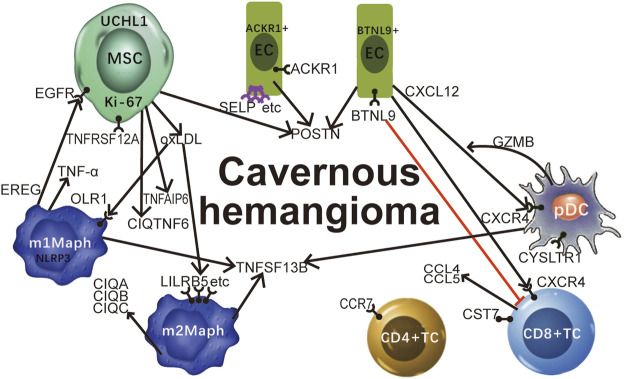
Featured genes and pathways in cavernous hemangiomas. Proinflammatory cytokines and related genes *TNF* (encoding TNF-α), *TNFSF13B*, *TNFRSF12A*, *TNFAIP6*, and *C1QTNF6* are significantly involved in the MSC-induced immune responses in cavernous hemangiomas; The MSCs in cavernous hemangioma exhibit characteristics of tumours, *e.g.,* high expression of *GAPDH* and very high expression of *MKI67* (encoding Ki-67). *UCHL1* can be used as a marker gene to detect embryonic MSCs at different apoptosis stages. The highly expressed *EREG* in M1-like macrophages interacted with the highly expressed *EGFR* in MSCs may cause MSC proliferation and the tumour progression through the binding of *EREG* to *EGFR*. MSCs induced the responses of ACKR1 positive endothelial cells (ACKR1+ECs) and BTNL9 positive endothelial cells (BTNL9+ECs). Adhesion molecules including *SELP*, *SELE*, and *VCAM1* were up-regulated leading to leukocyte transmigration across the endothelium to the site of inflammation. *POSTN* was expressed at very high levels in MSCs, high levels in the EC2 cluster, and medium levels in the BTNL9+EC and ACKR1+EC. *CCR7* has the potential to be a new marker to discriminate the CD4+T cells from the activated CD8+T cells. *CST7* was unexpectedly detected to be expressed at high levels in activated CD8+T cells, medium levels in NK cells, and barely detected in non-immune cells. CD8+T cells and NK cells may highly express *CCL5* for their infiltration in cavernous hemangiomas, independent on the tumour cell-derived CCL5-IFNG-CXCL9 process. The ligand-receptor interactions between CXCL12 and CXCR4 play an significant role in the immune responses in cavernous hemangioma. The highly expressed *BTNL9* in BTNL9+EC may cause checkpoint blockade through the binding of BTNL9 to T-cells. The highly co-expression of *CXCR4* and *GZMB* suggested that pDCs function for anti-tumour as CD8+T cells. We propose that OxLDL induces the OxLDL-OLR1-NLRP3 process in M1-like macrophages via the over-expression of *OLR1*, whereas OxLDL induces the OxLDL-SRs-C1q process in M2-like macrophages via the over-expression of many SR genes (*LILRB5*, etc) except *OLR1*. MSC: mesenchymal stem cell; OxLDL: oxidised low-density lipoprotein; SR: scavenger receptor; TNF: tumour necrosis factor.

As proinflammatory cytokines (*i.e.* IFN-γ and TNF-α) induce the apoptosis of MSCs, we examined the expression levels of the genes encoding INFs (interferons), the receptors of INFs, TNFSFs (tumour necrosis factor superfamily members), TNFRSFs (TNF receptor superfamily members), TNFAIPs (TNF alpha induced proteins) and C1QTNFs (C1q and TNF related proteins) and marker genes of cell apoptosis (*CASP1*-*10*, *BAX*, and *BCL2*) using our scRNA-seq data. The results (Supplementary File S3) included (Figure 4): 1) the *BAX/BCL2* ratio was markedly higher in the embryonic MSCs than other cells (Supplementary Figure S9), indicating the MSC apoptosis; 2) *IFNG* (encoding IFN-γ) was barely detected in all types of cells (Supplementary Figure S10); 3) *TNF* (encoding TNF-α) was markedly higher expressed in the M1-like macrophages and mast cells than other cells (Supplementary Figure S11), however, was only detected in 21% of M1-like macrophages and 23.4% of mast cells; 4) *TNFSF13B* was markedly higher expressed in DCs and macrophages than other cells (Supplementary Figure S11); and 5) *TNFRSF12A* (Supplementary Figure S12), *TNFAIP6* (Supplementary Figure S13), and *C1QTNF6* (Supplementary Figure S14) were markedly higher expressed in the embryonic MSCs than other cells. Based on the above results, we concluded that: 1) proinflammatory cytokines and related genes *TNF*, *TNFSF13B*, *TNFRSF12A*, *TNFAIP6*, and *C1QTNF6* are significantly involved in the MSC-induced immune responses in cavernous hemangiomas; 2) *UCHL1* is up-regulated in the embryonic MSC apoptosis induced by proinflammatory cytokines (*e.g., TNF* and *TNFSF13B*); and 3) *UCHL1* can be used as a marker gene to detect embryonic MSCs at different apoptosis stages.

### Two Clusters of Endothelial Cells

The EC1 and EC2 clusters contained 9.57% (1,032/10,784) and 21.08% (2,273/10,784) of the total cells, respectively. Among the five marker genes of EC1 (Table 1), two (*TFF3* and *MMRN1*) were highly expressed in LECs, whereas the other three (*EFNB2, RAPGEF5*, and *TIE1*) were highly expressed in EC2 (Figure 1B). *MKI67* (marker of Ki-67 proliferation) was expressed at a markedly higher level in EC2 than EC1. Most of *VEGF* and *FGF* genes (*HGF*, *VEGFA*, *VEGFB*, *FGF1*, *FGF9*, *FGF10*, *FGF11*, *FGF13*, *FGF14*, *FGF16*, *FGF18*, and *FGF22*) were expressed at markedly higher levels in EC2 than EC1, while only a few (*FGF2*, *FGF11*, and *FGF18*) were expressed at markedly lower levels in EC2 than EC1 and a few (*VEGFC*, *FGF5*, and *FGF13*) at similar levels (Supplementary Figure S15). The above results suggested that the cells of EC2, not EC1, were undergoing proliferation, since the VEGF proteins mainly induce proliferation of ECs. Such results were consistent with the phenomenon that “normal” vascular ECs can be observed in the lesions of cavernous hemangiomas by microscopic examination (**Introduction**). However, the cells of EC1 were not really “normal” (**As analyzed in the following paragraph**). Unexpectedly, *VEGFA* and *VEGFB* were detected to be expressed at markedly higher levels in the m1Maph and pDC clusters than other cells, respectively, which merit investigation in future studies.

Although the cells of EC1 were not undergoing proliferation, they were not really “normal” and may belong to immune-responsive ECs, transformed from or by embryonic MSCs, with markedly changed expression of the major histocompatibility complex (MHC) class I and II genes. MHC I genes are expressed by all nucleated cells, while the expression of MHC II genes is limited to antigen-presenting cells (APCs) (Mai et al., 2013). Professional APCs (*e.g.*, BCs, DCs, and macrophages) ubiquitously express MHC II, while cells such as ECs, which are not considered as APCs in the classical view, can induce MHC II expression in response to stimulation. Differential expression analysis (Supplementary File S2) between the EC1 and EC2 clusters revealed that almost all the MHC II genes (particularly, *HLA-F*, *HLA-DMA*, *HLA-DMB*, *HLA-DPA1*, *HLA-DPB1*, *HLA-DQA1*, *HLA-DQB1*, *HLA-DRA*, *HLA-DRB1*, and *HLA-DRB5*) were expressed at markedly higher levels in EC1 than EC2 (Supplementary Figure S16). Among other MHC II genes, *HLA-A*, *HLA-B*, *HLA-C*, and *HLA-E* were expressed at very high levels in all types of cells except the mast cells, whereas *HLA-DOA*, *HLA-DOB*, *HLA-DQA2*, and *HLA-DQB2* were barely detected (Avg_in ≤ 0.05) in the EC1 and EC2 clusters. Therefore, we concluded that EC1 is a cluster of immune-responsive ECs. Various factors and stimuli, including cytokines, can induce type I activation of ECs, a state of heightened responsiveness (Mai et al., 2013). The type I activation is a rapid response that is mediated by the binding of ligands to the extracellular domains of heterotrimeric G protein-coupled receptors (GPCRs). All the detected GPCRs in the cavernous hemangioma (*ADRA1D*, *ADRA2B*, *ADRA2C*, *AVPR2*, *CNR1*, *GALR1*, *GPR20*, *LPAR4*, *OXTR*, *P2RY2*, *TACR1*, *CYSLTR1*, *TAS1R1*, *TAS2R43*, *MAS1*, *GPR156*, *P2RY8*, *GPR52*, and *GPR85*) were expressed at markedly higher levels in EC1 than EC2 (Supplementary Figure S17). The above results indicated that the type I activation was induced in EC1. Compared to the type I activation, the type II activation of ECs is a relatively slower response that depends on new gene expression but delivers a more sustained inflammatory response (Mai et al., 2013). The type II activation of ECs in cavernous hemangiomas merits investigation in future studies.

EC1 was further clustered into two subtypes: *ACKR1* positive endothelial cell (ACKR1+EC) and *BTNL9* positive endothelial cell (BTNL9+EC), including 664 and 368 cells, respectively. *ACKR1*, *SELP*, *SELE*, *VCAM1,* and *CADM3* were expressed at markedly higher levels in ACKR1+EC than BTNL9+EC (Supplementary Figure S18). Among the five genes, *ACKR1* encodes a glycosylated membrane protein as a non-specific receptor for several chemokines and may regulate chemokine bioavailability and consequently, leukocyte recruitment through two distinct mechanisms; *CADM3* encodes a calcium-independent cell-cell adhesion protein that can form homodimers or heterodimers with other nectin proteins; the three other genes (*SELP*, *SELE*, and *VCAM1*) encode adhesion molecules that induce the adhesion of immune cells, such as leukocytes, to the endothelium and facilitate transmigration to underlying tissues (Mai et al., 2013). However, another adhesion molecule *ICAM1* was not detected in all types of cells. The expression of genes encoding adhesion molecules can be induced by oxidised low-density lipoprotein (OxLDL) (Mai et al., 2013). OxLDL induces cellular processes through scavenger receptors (SRs) (Alquraini and El Khoury, 2020), which are a large family of structurally diverse receptors recognizing a range of ligands including modified LDLs, selected polyanionic ligands, and microbial structures. Then, we found 10 known SR genes (*OLR1*, *MRC1*, *STAB1*, *CD163*, *MSR1*, *CD36*, *CD68*, *CXCL16, CLEC7A*, and *LILRB5*) by searching top 100 DE genes from the gene-expression signatures of EC1, m1Maph, and m2Maph. However, almost all of them were expressed at very low levels in both EC1 and EC2 clusters except *STAB1* and *CD36* (Supplementary Figure S19). Among the 10 SR genes, only *CD36* was expressed at a very high level in EC1, but exhibited a lower average expression level in ACKR1+EC than in BTNL9+EC. The above results suggested that the up-regulated expression of adhesion molecules in ACKR1+EC may be induced by OxLDL through *CD36*, but more likely through other SRs. However, it is not clear why OxLDL did not induce expression of the genes encoding adhesion molecules SELP, SELE, VCAM1, and ICAM1 in BTNL9+EC.

*BTNL9* and *CXCL12* can be used to characterize BTNL9+EC, as they were expressed at markedly higher levels in BTNL9+EC than ACKR1+EC (Supplementary Figure S18). As a member of the BTN/MOG Ig-superfamily, *BTNL9* is expressed in a variety of tissues in humans and mice, and functions as a negative regulator of immune cell activation. Recombinant BTNL9–Fc has been demonstrated to bind to many immune cells, including macrophages, T, B, and dendritic cells. In particular, BTNL9 has been reported to inhibit CD8^+^ T-cell proliferation (Arnett and Viney, 2014), however, its receptors are still unknown. According to previous studies, *CXCL12* encodes a stromal cell-derived alpha chemokine member of the intercrine family and stimulates the migration of monocytes and T-lymphocytes through its receptors encoded by *CXCR4* and *ACKR3* (Supplementary Figure S18). In the present study, *CXCL12* was detected to be significant lower expressed in the immune cells than the non-immune cells, particularly fibroblast and EC1, while *CXCR4* was detected to be significant lower expressed in the non-immune cells than the immune cells, particularly pDC and CD8+TC. Unexpectedly, *ACKR3* was barely detected (Avg_in ≤ 0.05) to be expressed in the immune cells except m1Maph, and very lowly expressed in the non-immune cells except fibroblast. The above results suggested that (Figure 4): 1) the ligand-receptor interactions between CXCL12 and CXCR4 play a significant role in the immune responses in cavernous hemangiomas; and 2) the high expression of *BTNL9* may cause checkpoint blockade in BTNL9+EC through the binding of BTNL9 to T-cells (Figure 4).

### Two Subsets of Macrophages

Two subsets of macrophages were intensively investigated. A number of previous studies have revealed that a considerable degree of monocyte–macrophage heterogeneity exists when various marker genes are used to identify macrophage subsets (Mihail and Christian, 2011). An over-simplified generalisation of this concept recognises M1 and M2 macrophages. M1 macrophages have inflammatory and anti-tumour properties, while M2 macrophages have anti-inflammatory and tumour-promoting abilities (Han et al., 2019). M1 macrophages mainly secrete proteins encoded by interleukin 12A (*IL12A*), interleukin 12 B (*IL12B*), and tumour necrosis factor (*TNF*), whereas M2 macrophages typically produce proteins encoded by interleukin 10 (*IL10*), interleukin 1 receptor antagonist (*IL1RN*), and interleukin 1 receptor type 2 (*IL1R2*) (Frumento et al., 2006). The published marker genes for M1 macrophages include *IL1B* and *NFKB1*, while those for M2 macrophages include *MERTK*, *MRC1*, *STAB1*, and *CD163*. Among the above 12 marker genes (Supplementary Figure S19), no single one can be used to clearly discriminate M1 from M2 macrophages. For instance, 65.84% (607/922) of the macrophage type-1 cells and 88.18% (1,134/1,286) of the macrophage type-2 cells expressed *CD163* at similar average levels. Another instance is *MERTK* that was expressed at very high levels in both macrophage type 1 and type 2 cells. Then, we identified the macrophage type-1 and type-2 clusters as M1-like and M2-like macrophage (m1Maph and m2Maph) clusters, using the five marker genes (Table 1) together, respectively.

In m1Maph, 66.3, 83.4, 64.5, 82.5, and 71.3% of the cells expressed the five marker genes *OLR1*, *EREG*, *BCL2A1*, *SLC11A1*, and *NLRP3*, respectively (Figure 1B). According to annotations from the GeneCards database (Marilyn et al., 2002), *OLR1* (also named *LOX-1*)*,* as a notable SR gene, is up-regulated in responses to stimulation by OxLDL, proinflammatory cytokines, and proatherogenic factors such as angiotensin II in ECs. According to a previous study (Wen et al., 2020), OxLDL-induced NLRP3 inflammasome activation in macrophages plays a vital role in atherogenesis. Thus, the detection of *OLR1* and *NLRP3* at very high expression levels revealed that OxLDL induced NLRP3 inflammasome activation in m1Maph through *OLR1* (named as the OxLDL-OLR1*-*NLRP3 process). Although the nine other SR genes (*MRC1*, *STAB1*, *CD163*, *MSR1*, *CD36*, *CD68*, *CXCL16*, *CLEC7A*, and *LILRB5*) were also expressed at very high levels in m1Maph, they may not be involved in or contribute slightly to the OxLDL-NLRP3 process, as they were very highly expressed with markedly lower co-expression of *OLR1* and *NLRP3* in m2Maph. Among all types of cells in the cavernous hemangioma, only m1Maph highly expressed *EREG*, which provided a deeper understanding of the origin and functions of epiregulin in the immune responses. According to annotations from the GeneCards database (Marilyn et al., 2002), the protein, epiregulin, encoded by *EREG* is a ligand of *EGFR* and structurally related erb-b2 receptor tyrosine kinase 4 (*ERBB4*). According to previous studies, *EREG* may be involved in a wide range of biological processes, including inflammation, wound healing, oocyte maturation, and cell proliferation. In particular, *EREG* promotes cancer progression in various human tissues. By single-cell transcriptome analysis, a previous study (Ma et al., 2021) has revealed that *EREG* is predominantly expressed in macrophages in the TME and induces EGFR-tyrosine kinase inhibitor (TKI) resistance in the treatment of non-small cell lung cancer (NSCLC) by preventing apoptosis through the EGFR/ErbB2 heterodimer. In the present study, *EGFR* was expressed at high levels in fibroblasts, SMCs and MSCs, while *ERBB4* was barely detected in all types of cells. The above results suggested that 1) m1Maph may promote the tumour progression through the binding of EREG to EGFR, challenging the current theory that M1 macrophages have inflammatory and anti-tumour properties. 2) *EGFR* inhibitors can be used to treat cavernous hemangiomas.

In m2Maph, 66.3, 72.6, 83.3, 78, and 85% of the cells expressed the five marker genes *FOLR2*, *LILRB5*, *C1QC*, *MS4A4A*, and *C1QB*, respectively (Figure 1B). According to previous studies, the serum complement subcomponent, C1q, is composed of 18 polypeptide chains which include six A-chains, six B-chains, and six C-chains, encoded by complement C1q A chain (*C1QA*), complement C1q B chain (*C1QB*), and complement C1q C chain (*C1QC*) genes. The C1q protein enhances the survival and efferocytosis of macrophage foam cells, which is thought to be induced by LDL, including OxLDL or minimally modified LDL (mmLDL) (Shashkin et al., 2005). In addition, understanding the molecular mechanisms underlying OxLDL- and mmLDL-induced macrophage foam cell formation is of fundamental importance for atherosclerosis and cardiovascular disease. Among all types of cells in the cavernous hemangioma, only m2Maph highly expressed *C1QA*, *C1Q*B, and *C1QC*, which provided a deeper understanding of the origin and functions of C1q in the immune responses. However, whether m2Maph included macrophage foam cells is unknown. Another previous study (Chen et al., 2021) has reported that the expression levels of *C1QA*, *C1Q*B, and *C1QC* are positively related to M1, M2 macrophages and CD8^+^ cells, and negatively correlated with M0 macrophages, whereas our results demonstrated that *C1QA*, *C1QB*, and *C1QC* were expressed at very high levels in m2Maph, very low levels in m1Maph and barely detected in activated CD8+T cells. The detection of highly expressed *LILRB5*, *C1QA*, *C1QB*, and *C1QC* suggested that OxLDL induced the inflammatory activation of C1q (named as the OxLDL-LILRB5-C1q process) in m2Maph through *LILRB5*. Although the nine other SR genes (*OLR1*, *MRC1*, *STAB1*, *CD163*, *MSR1*, *CD36*, *CD68*, *CXCL16*, and *CLEC7A*) were also expressed at very high levels in m2Maph, they may not be involved in or contribute minimally to the OxLDL-LILRB5-C1q process, as they were very highly expressed with markedly lower co-expression of *LILRB5*, *C1QA*, *C1QB*, and *C1QC* in m1Maph. Based on the above results, we proposed that OxLDL induces the OxLDL-OLR1-NLRP3 process in m1Maph and the OxLDL-LILRB5-C1q process in m2Maph (Figure 4).

### T Lymphocytes and NKCs

The TC1, TC2, and TC3 clusters containing 5.53% (596/10,784), 2.85% (307/10,784), and 1.86% (201/10,784) of the total cells, respectively, were further identified as CD4+TC, CD8+TC, and NKC clusters (**Described as above**). The cells in the three clusters were entangled, as CD4+TC, CD8+TC, and NKC expressed some common genes without significant differences. For instance, both CD8+TC and NKC expressed *NKG7* (a marker gene of NK cells) at very high levels. Therefore, we compared the expression levels of more relevant genes to confirm the cell types of the three clusters. The NK cells were confirmed based on the following evidences (Supplementary Figure S20): 1) the average expression levels of *CD3D, CD3E, CD3G, CD4, CD8A*, and *CD8B* (marker genes of T cells) in NKC were lower than 5% of those in CD4+TC and CD8+TC, respectively; 2) the average expression level of *NKG7* in NKC was approximately 92-fold higher than that in CD4+TC; and 3) the average expression levels of *GZMA, GZMB, GZMH, GZMK, GZMM, CRTAM,* and *GNLY* in NKC were approximately 12.3, 51, 4.56, 102.2, 1, 11.2, and 151-fold higher than those in CD4+TC, respectively. Although both CD4+TC and CD8+TC expressed *CD8B* (a marker gene of CD8+T cells) at a similar level, the cells in CD4+TC could still be clearly discriminated from the cells in CD8+TC based on the following evidences: 1) the average expression level of *CD4* in CD4+TC was higher than 5-fold of that in CD8+TC; 2) the average expression level of *CD8A* in CD8+TC was higher than 4-fold of that in CD4+TC; 3) the average expression levels of *NKG7, GZMA, GZMB, GZMH, GZMK, GZMM, CRTAM,* and *GNLY* in CD4+TC were markedly lower than those in NKC and CD8+TC; and 4) the average expression levels of *GZMA, GZMH, GZMK*, and *GZMM* in CD8+TC were higher than 2-fold of those in CD4+TC, while the average expression levels of *GZMB, CRTAM,* and *GNLY* in CD8+TC were only approximately 53.1, 35.7, and 15% of those in NKC, respectively. By further analysis of gene-expression signatures of CD4+TC, CD8+TC, and NKC, we obtained two new results (Figure 4): 1) *CCR7* was expressed at a markedly higher level than *CD4, CD8A*, and *CD8B*, and the average expression level of *CCR7* in CD4+TC was higher than 38-fold of that in CD8+TC and 20-fold of that in NKC; and 2) *CST7* was detected to be significant lower expressed in the non-immune cells than the immune cells, particularly CD8+TC and NKC. However, the expression of *CST7* has been detected in various human cancer cell lines established from malignant tumours. According to annotations from the GeneCards database (Marilyn et al., 2002), *CST7* encodes a glycosylated cysteine protease inhibitor with a putative role in immune regulation through the inhibition of a unique target in the hematopoietic system. Based on the above results, we concluded that *CCR7* has the potential to be a new marker to discriminate CD4+T cells from activated CD8+T cells. The specific functions of highly expressed *CST7* in CD8+T and NK cells merit investigation in future studies.

Among the top 20 highly expressed genes ranked by their average (the arithmetic mean) expression levels, both *CCL4* and *CCL5* encode chemokine ligands that have chemokinetic and inflammatory functions by binding to their receptors encoded by *CCR5*. According to previous studies of *CCL4* (Mukaida et al., 2020) *and CCL5* (Dangaj et al., 2019), the over-expression of *CCL5* is associated with CD8+T cell infiltration in solid tumours, while *CCL4* can promote tumour development and progression by recruiting regulatory T cells and pro-tumorigenic macrophages, and acting on other resident cells (*e.g.*, fibroblasts and endothelial cells) present in the tumour microenvironment (TME) to facilitate their pro-tumorigenic capacities. In some situations, *CCL4* can enhance tumour immunity by recruiting cytolytic lymphocytes and macrophages with phagocytic ability. Furthermore, the previous study (Dangaj et al., 2019) has reported that the T cell infiltration requires tumour cell-derived *CCL5*, and this process is amplified by IFN-γ-inducible, myeloid cell-secreted *CXCL9*. As IFN-γ is encoded by *IFNG*, we named this amplification process as tumour cell-derived CCL5-IFNG-CXCL9 process. In the previous study (Dangaj et al., 2019), the co-expression of *CCL5* and *CXCL9* has been detected to reveal immunoreactive tumours with prolonged survival and response to PD-1 inhibition. By examining the expression levels of *CCL4*, *CCL5*, *CCR5*, *IFNG*, *CXCL9*, and *PDCD1* (well-known as *PD-1*), we found that (Supplementary Figure S21): 1) *CCL4* was detected to be significant lower expressed in the non-immune cells than the immune cells, particularly CD8+TC, NKC and m2Maph; 2) *CCL5* was expressed at the highest level in CD8+TC and the second highest level in NKC; 3) *CCR5* was very lowly expressed in CD8+TC and m1Maph and barely detected in other cells and; 4) *IFNG* and *CXCL9* were barely detected in all types of cells; and 5) *PDCD1* (well-known as *PD-1*) was only barely detected in CD4+TC and CD8+TC. Based on the above results, we concluded that the tumour cell-derived CCL5-IFNG-CXCL9 process was not induced in cavernous hemangiomas. The specific functions of highly expressed *CCL4* and *CCL5* in CD8+T and NK cells merit investigation in future studies (Figure 4).

### Other Cells

For the other five clusters, LECs, BCs, mDCs, pDCs, and CLEC9A + DCs contained 0.35% (38/10784), 1.1% (119/10784), 2.47% (266/10784), 0.33% (36/10784), and 0.44% (47/10784) of the total cells, respectively. Differential expression analysis (**Materials and Methods**) between cells inside and outside each cluster was performed to produce a gene-expression signature including all DE genes (Supplementary File S2). The top five DE genes were selected as a combination of marker genes for each cell type (Table 1). Of note, *CXCR4*, *GZMB*, and *CYSLTR1* were detected to be expressed in the pDC cluster at very high levels (Figure 4). The high co-expression of *CXCR4* and *GZMB* suggested that pDCs function for anti-tumour as CD8+T cells in cavernous hemangiomas. Although the proportion of pDCs was markedly lower than that of CD8+T cells, their contribution to anti-tumour activity may complement the loss caused by checkpoint blockade in CD8+T cells. *CYSLTR1* encodes a protein that is a second receptor for cysteinyl leukotrienes and is thought to be the main receptor mediating cysteinyl leukotriene receptor smooth-muscle contraction and inflammatory cell cytokine production in asthma. However, the specific functions of highly expressed *CYSLTR1* in pDCs remain unknown.

## Conclusion and Discussion

In the present study, we identified 16 cell types in a cavernous hemangioma using scRNA-seq and provided a gene-expression signature (Supplementary File S2) and a combination of five marker genes (Table 1) to each cell type. These gene-expression signatures can be used as references for the exact identification of these cell types in future studies. The main contribution of the present study is that we discovered a large number (5.66% of the total cells) of embryonic MSCs in the cavernous hemangioma. According to the current theory, hemangiomas originate from neogenesis or revival of dormant embryonic angioblasts and arise through hormonally driven vessel growth (**Introduction**). However, we ruled out the possibility that the MSCs were transformed from angioblasts. We also ruled out the possibilities that the MSCs were pericyte derivatives, CAFs, bone marrow-derived MSCs or generated via EMT. Therefore, we proposed that cavernous hemangiomas may originate embryonic MSCs. Different types of hemangiomas probable originate from embryonic MSCs in different tissues or under different conditions. Further analysis of the embryonic MSCs revealed that:1) proinflammatory cytokines and related genes *TNF*, *TNFSF13B*, *TNFRSF12A*, *TNFAIP6*, and *C1QTNF6* are significantly involved in the MSC-induced immune responses in cavernous hemangiomas; 2) *UCHL1* is up-regulated in the embryonic MSC apoptosis induced by proinflammatory cytokines (*e.g.*, *TNF* and *TNFSF13B*); 3) the UCHL1-induced apoptosis of MSCs may play an important role in the MSC-induced immune responses in cavernous hemangiomas; and 4) *UCHL1* can be used as a marker gene to detect embryonic MSCs at different apoptosis stages. In addition to MSCs, ECs, macrophages, T lymphocytes and NKCs were intensively investigated, revealing the genes and pathways featured in cavernous hemangiomas (Figure 4).

Our on coming research work will be conducted, following two clues: identification of additional embryonic MSCs-caused cancers/tumours and investigation of UCHL1-induced apoptosis in other types of cancers/tumours. To achieve the first goal, it requires the use of the gene expression signature from the present study as a reference to identify the embryonic MSCs in more other types of cancers/tumours with the combination of different machine learning methods on public scRNA-seq data. The discovery of UCHL1-induced apoptosis of MSCs in cavernous hemangiomas preliminarily explained the benign nature of cavernous hemangiomas. However, the molecular mechanism underlying the UCHL1-induced apoptosis of MSCs is still unknown. We proposed that proinflammatory cytokines (*e.g.*, *TNF* and *TNFSF13B*) triggered the UCHL1-induced apoptosis through their receptors (*e.g.*, *TNFRSF12A*) and this process can be regulated by other proteins (*e.g.*, *TNFAIP6* and *C1QTNF6*). The better understanding of the MSC-induced immune responses in benign tumours helps to guide future investigation and treatment of embryonic MSC-caused tumours. For example, *POSTN* and *UCHL1* can be used to control the differentiation, development or apoptosis of transplanted MSCs. Proinflammatory cytokines (*e.g.*, IFN-γ and TNF-α) can be used to treat hemangiomas or other embryonic MSC-caused tumours via the UCHL1-induced apoptosis of MSCs. As TNFAIP6 can be induced by both IFN-γ and TNF-α, it can be used as a marker to evaluate the treatment.

## Materials and Methods

### 10x Genomics Library Preparation and Sequencing

A piece of tissue in the centre of the tumour (**Results**) was digested for 0.5 h at 37°C in the enzyme solution (Enzyme H, R and A) using a gentleMACS Dissociator, following the manufacturer’s instruction. The single-cell suspension was filtered with a 40-μm-diameter cell strainer (FALCON, United States), then washed twice with RPMI1640 wash buffer at 4°C. Cell viability was determined by trypan blue staining with TC20 automated cell counter (BioRad, United States). The ratio of viable cells in single-cell suspension was more than 85%. The single-cell suspension was adjusted to have a concentration of 700–1,200 cells/μl, and then processed to generate 10x libraries with the Chromium Single Cell 3’ Reagent Kits v3 CG000183 (10x Genomics, United States) following the manufacturer’s instruction. These libraries were sequenced by an Illumina NovaSeq 6,000 sequencer, producing 542,088,397 pairs of 150-bp reads (∼163 Gbp raw scRNA-seq data).

### ScRNA-Seq Data Processing

Using the software Cell Ranger v4.0.0, we aligned 542,088,397 read2 sequences (∼) to the human genome GRCh38, generating an UMI-count matrix (33,694 genes × 6,794,880 cell barcodes). The cell-calling algorithm in Cell Ranger was used to identify 12,018 cells from the 6,794,880 cell barcodes. Then, a total of 10,784 cells and 22,023 genes were retained with a median of 2,084 genes per cell after quality control (QC) filtering using the following parameters: 1) genes detected in <3 cells were excluded; 2) cells with <200 genes were excluded; 3) cells with >30% mitochondrial RNA UMI counts or >5% hemoglobin RNA UMI counts were excluded; 4) 982 doublet artifacts were removed with DoubletFinder. Finally, a 22,010 × 10,784 matrix and a 13 × 10,784 matrix were produced to represent expression levels of nuclear and mitochondrial genes, respectively. The data including two matrices is available at the NCBI GEO database under the accession number GSE188515.

The R package Seurat v4.1.0 and other R packages (*e.g.,* ggplot2) were used for scRNA-seq data analysis on R v4.1.3 (Gao et al., 2014). Each column of the nuclear UMI-count matrix (22,010 × 10,784) was normalized using the NormalizeData function by dividing the sum of each column, then multiplying by a scale factor of 10,000 and taking its natural-log transformed value. We selected 2000 highly variable genes on the basis of the average expression and dispersion per gene using the FindVariableFeatures function. After data scaling, principal component analysis (PCA) was performed on the 2000 highly variable genes (not the total 22,010 genes) using the RunPCA function. The top 50 principal components were chosen from 100 calculated principal components for cell clustering using the FindNeighbors and FindClusters functions with parameters (algorithm = 4, resolution = 0.4). The Uniform Manifold Approximation and Projection (UMAP) method was used on the PCA&clustering result to show all the clusters.

### Identification of Cell Types and Selection of Marker Genes

For each cluster, differential expression analysis between cells inside and outside the cluster was performed to produce a gene-expression signature using the R package DESeq2. We used the function (scran:computeSumFactors) for scRNA-seq data analysis in DESeq2 for the normalization of the nuclear UMI-count matrix (22,010 × 10,784), which is different from the default normalization method in DESeq2 or that in Seurat. Then, we run the main function DESeq with the following parameters (test = “LRT”, fitType = “glmGamPoi”, minReplicatesForReplace = Inf, useT = TRUE, minmu = 1e-6). Genes with expression levels below 10% in both of the two groups of cells, were filtered out. For each cluster, a gene-expression signature includes all differentially expressed (DE) genes meeting two criteria (adjusted *p*-value ≤ 0.01 and LFCio ≥ 2). LFCio is the 2-based log-transformed fold change (*i.e.*,“log2FoldChange” calculated by DESeq2) between the average (arithmetic mean) expression levels of a gene inside and that outside a cluster, and Avg_in is the average expression level of a gene in the cluster. The identification of each cluster as a specific cell type took two-steps (the rough and exact identification). Firstly, each cluster was roughly identified by comparing several selected DE genes to known marker genes. As for the known marker genes, 1) most of them were used according to records in an online database (http://biocc.hrbmu.edu.cn/CellMarker/) by Harbin Medical University; and 2) a few were used according to records in published papers, *e.g.*, LINC00926 (Zhang et al., 2018).

Then, each cluster was exactly identified by further analysis of its gene-expression signature. GO and pathway annotation with analysis were performed using the Metascape website (https://metascape.org/gp) (Zhou et al., 2019). The top five DE genes was selected as a combination of marker genes from the gene-expression signature for each cluster (Table 1), meeting the following criteria: 1) the percentage of cells that expressed the gene inside the cluster (PCTin) > 60%; and 2) the ratio between the percentage of cells that expressed the gene inside (PCTin) and outside the cluster (PCTout) is ranked in the top five. As LFCio or the ratio between PCTin and PCTout can only be used to evaluate the identification of a cell type by a single marker gene, we designed another metric UICC to evaluate the representation of a cell type by a combination of marker genes. The cardinal number of the union set of cells expressed marker genes is divided by the number of all cells in a cluster to calculate the union coverage of a cluster (UCC). The cardinal number of the intersection set of cells expressed marker genes is divided by the number of all cells in a cluster to calculate the intersection coverage of a cluster (ICC). To balance UCC and ICC, we designed UICC, which is calculated by multiplying UCC by ICC.

## Data Availability

The original contributions presented in the study are included in the article/Supplementary Material, further inquiries can be directed to the corresponding authors. Finally, a 22,010 × 10,784 matrix and a 13 × 10,784 matrix were produced to represent expression levels of nuclear and mitochondrial genes, respectively. The data including two matrices is available at the NCBI GEO database under the accession number GSE188515.
