# Supplementary material for: Single-Cell Transcriptome Analysis Reveals Mesenchymal Stem Cells in Cavernous Hemangioma
Source: Front Cell Dev Biol. 2022 Jul 5;10:916045. doi: 10.3389/fcell.2022.916045 (PMC9294370; doi:10.3389/fcell.2022.916045)
Supplement: Supplementary file 1 [file DataSheet3.ZIP › s3.docx]

Supplementary 3

Fibroblast MSC


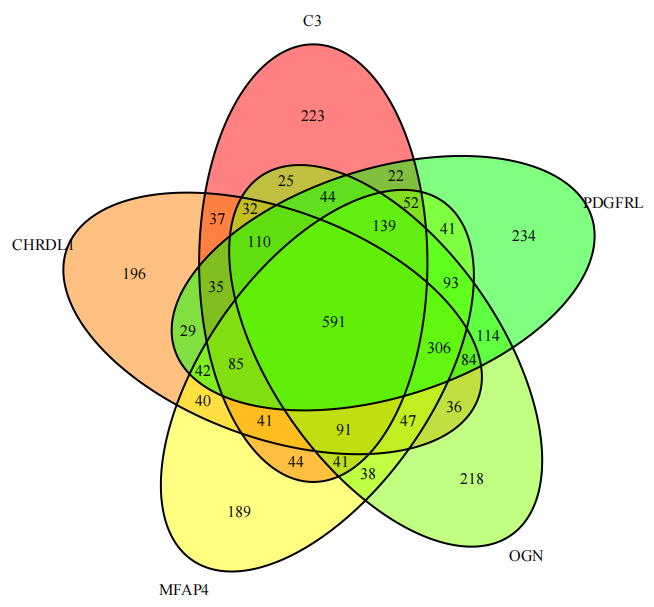

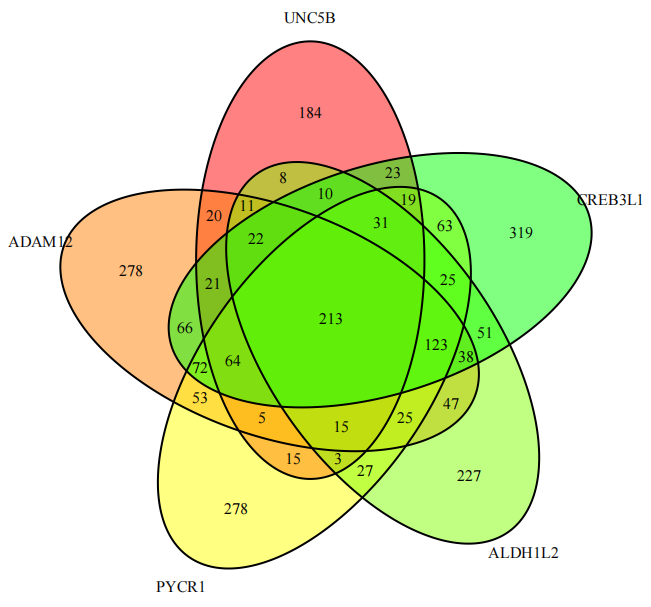


SMC EC1


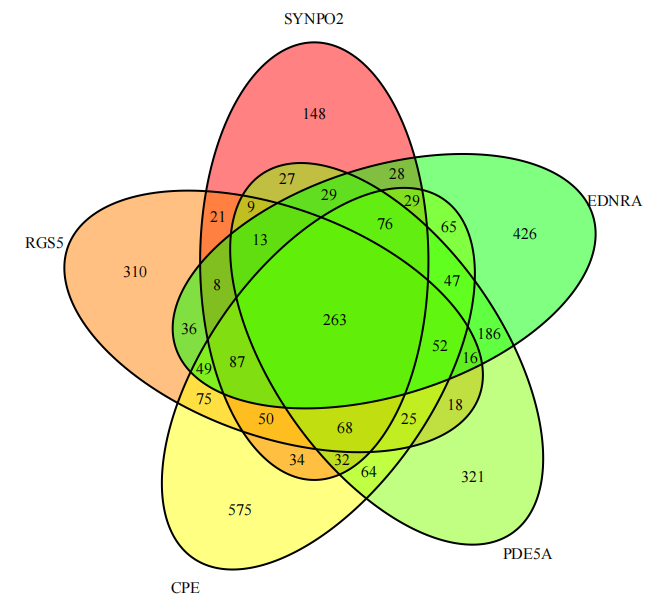

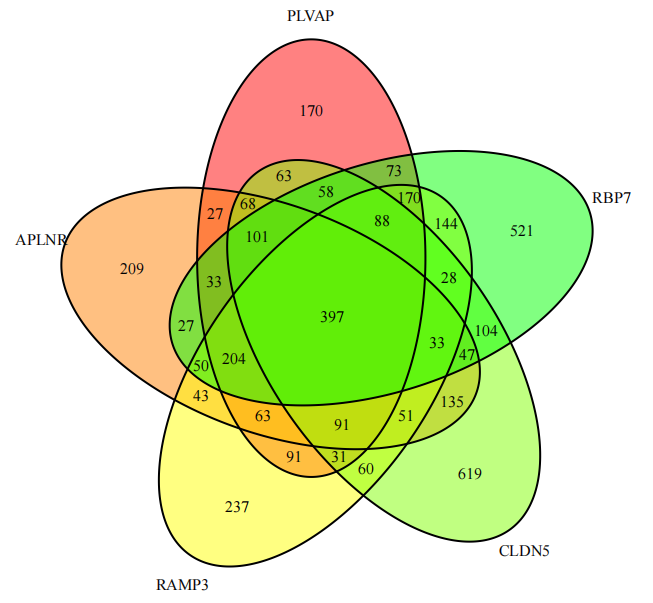


EC2 LEC


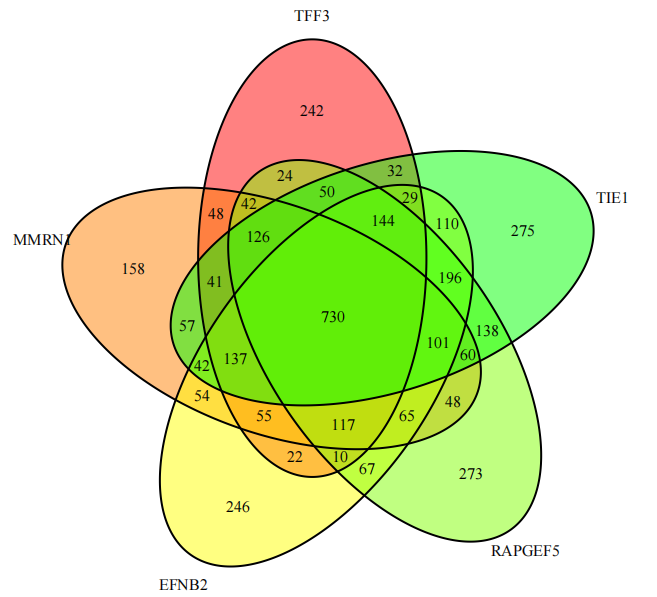

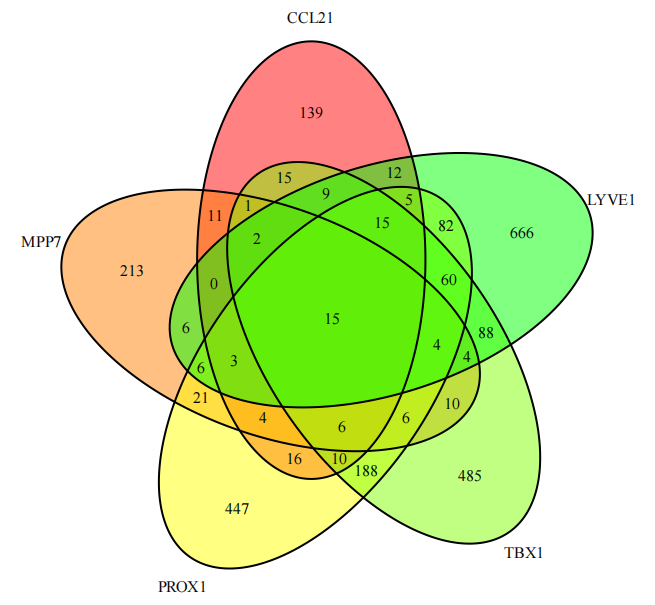


CD4+TC CD8+TC


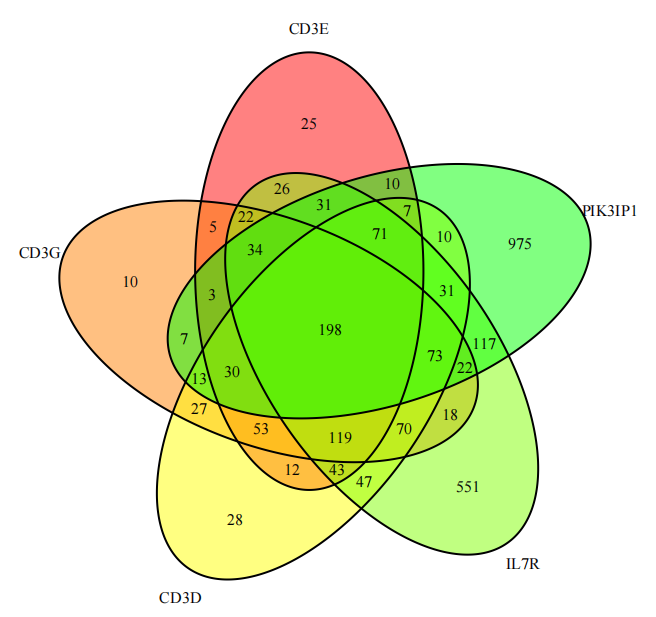

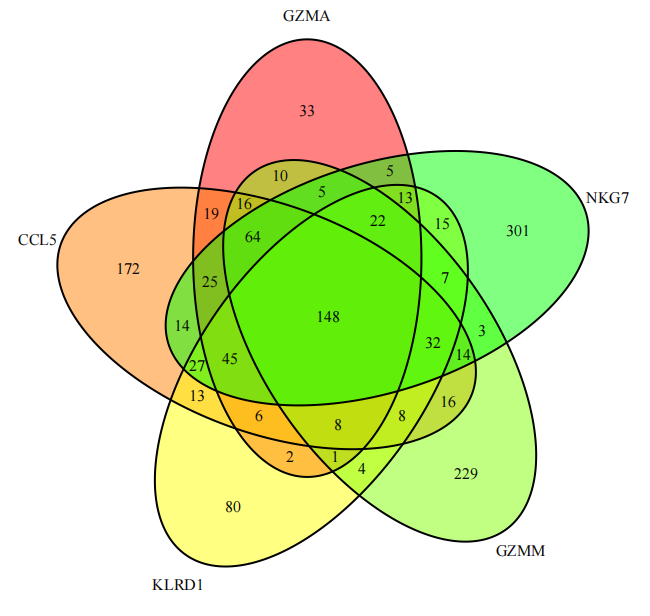


NKC BC


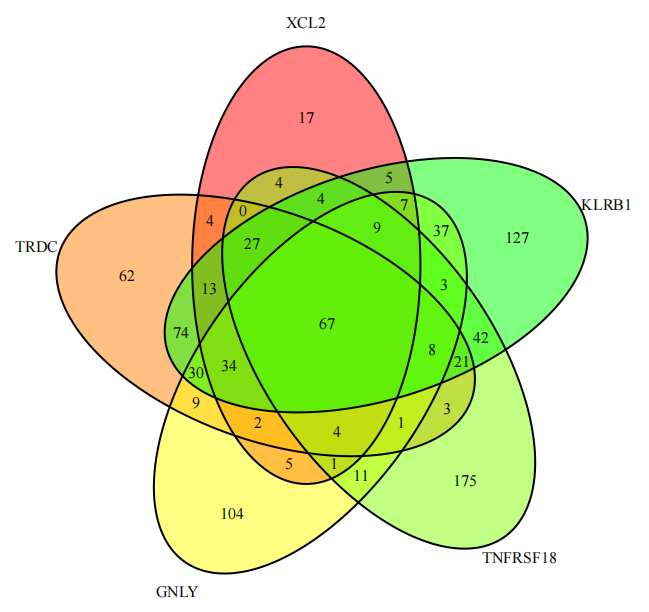

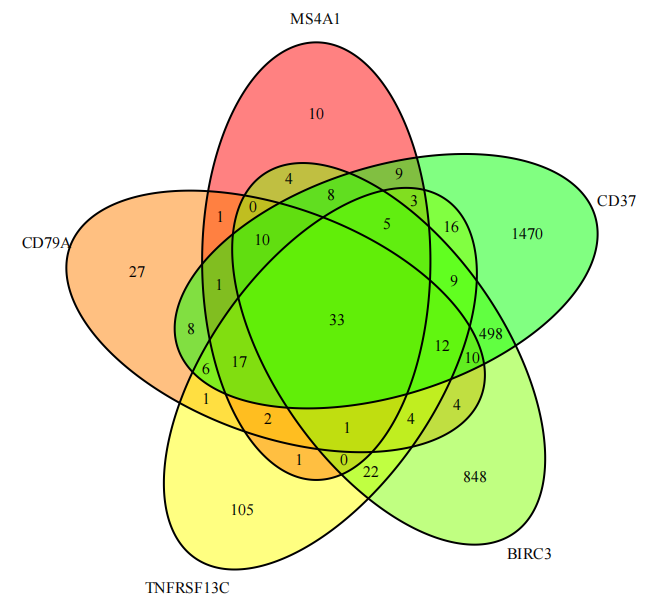


Mast mDC


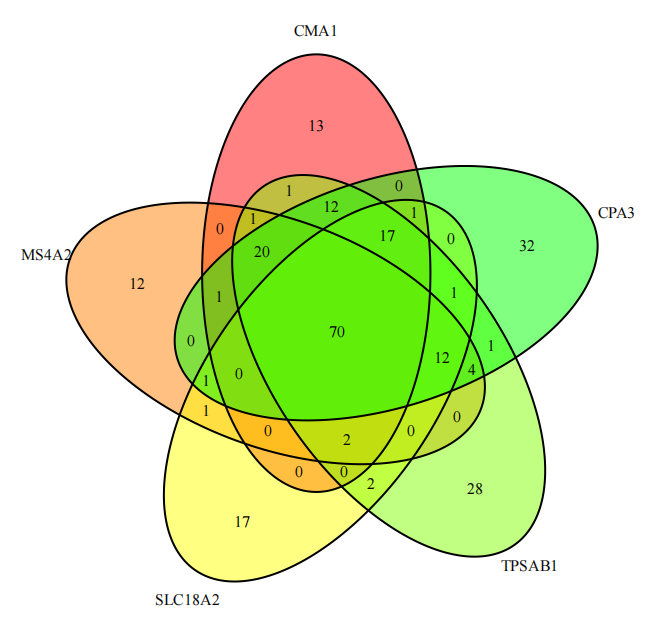

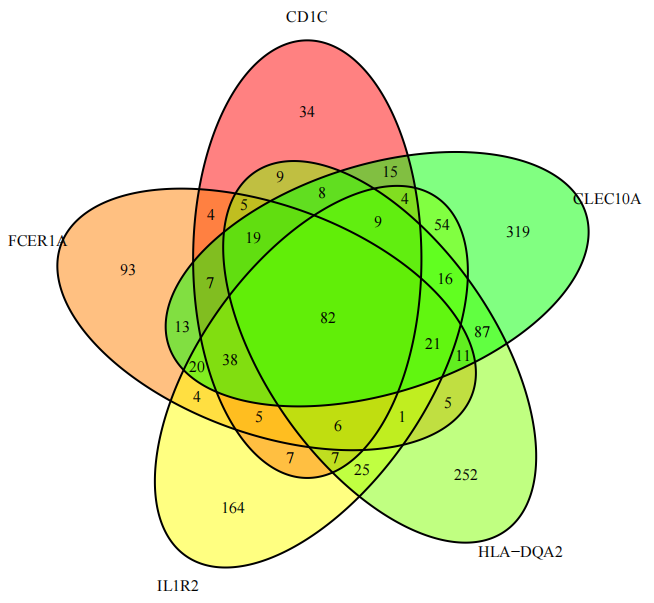


pDC CLEC9A+DC


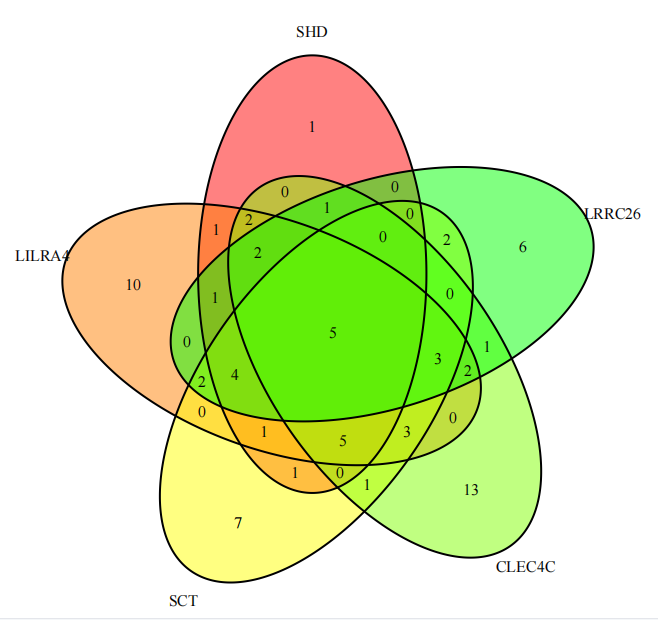

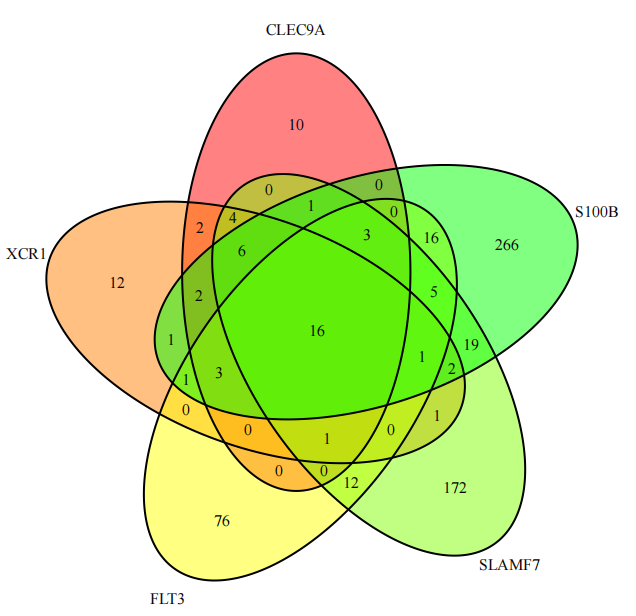


m1Maph m2Maph


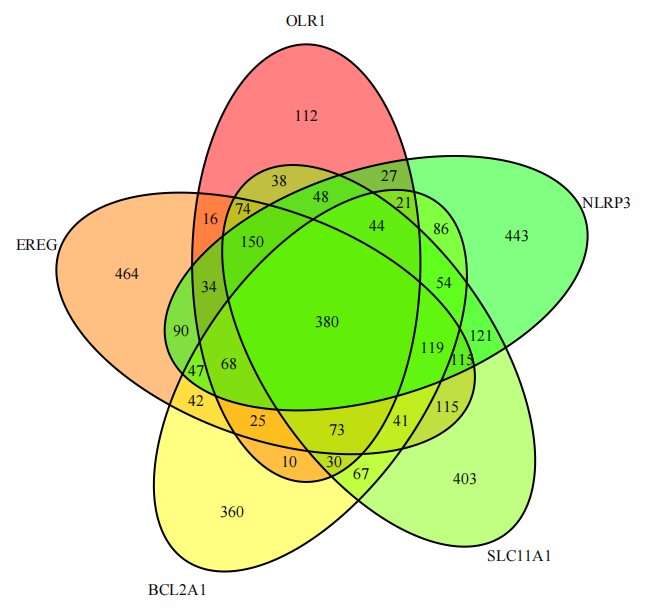

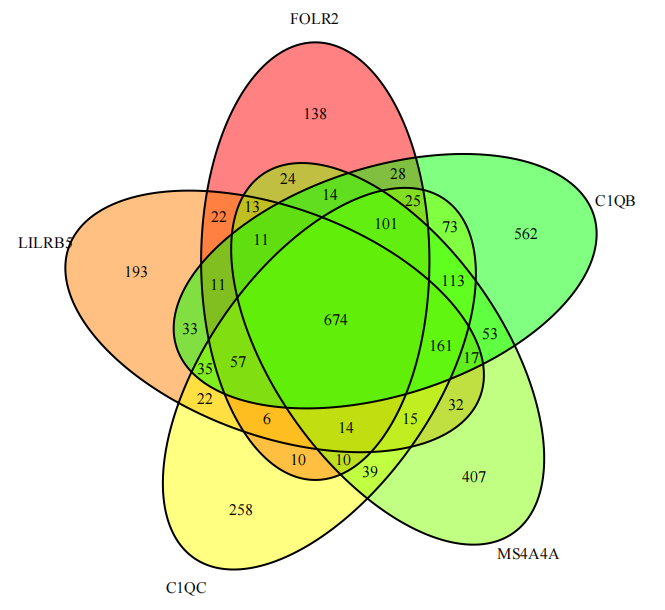


**Fig S4. Venn diagrams of** **marker genes**

The representation of a cell type by the combination of marker genes were also showed in Venn diagrams, which can be seen in a small-size figure (**Fig 1C**). MSC: mesenchymal stem cell, SMC: smooth muscle cell, EC1: endothelial cell type 1, EC2: endothelial cell type 2, LEC: lymphatic endothelial cell, CD4+TC: CD4 positive T cell, CD8+TC: CD8 positive T cell, NKC: natural killer cell, BC: B cell, mDC: monocyte derived dendritic cell, pDC: plasmacytoid dendritic cell, CLEC9A+DC: CLEC9A positive dendritic cell, m1Maph: M1-like macrophage and m2Maph: M2-like macrophage.


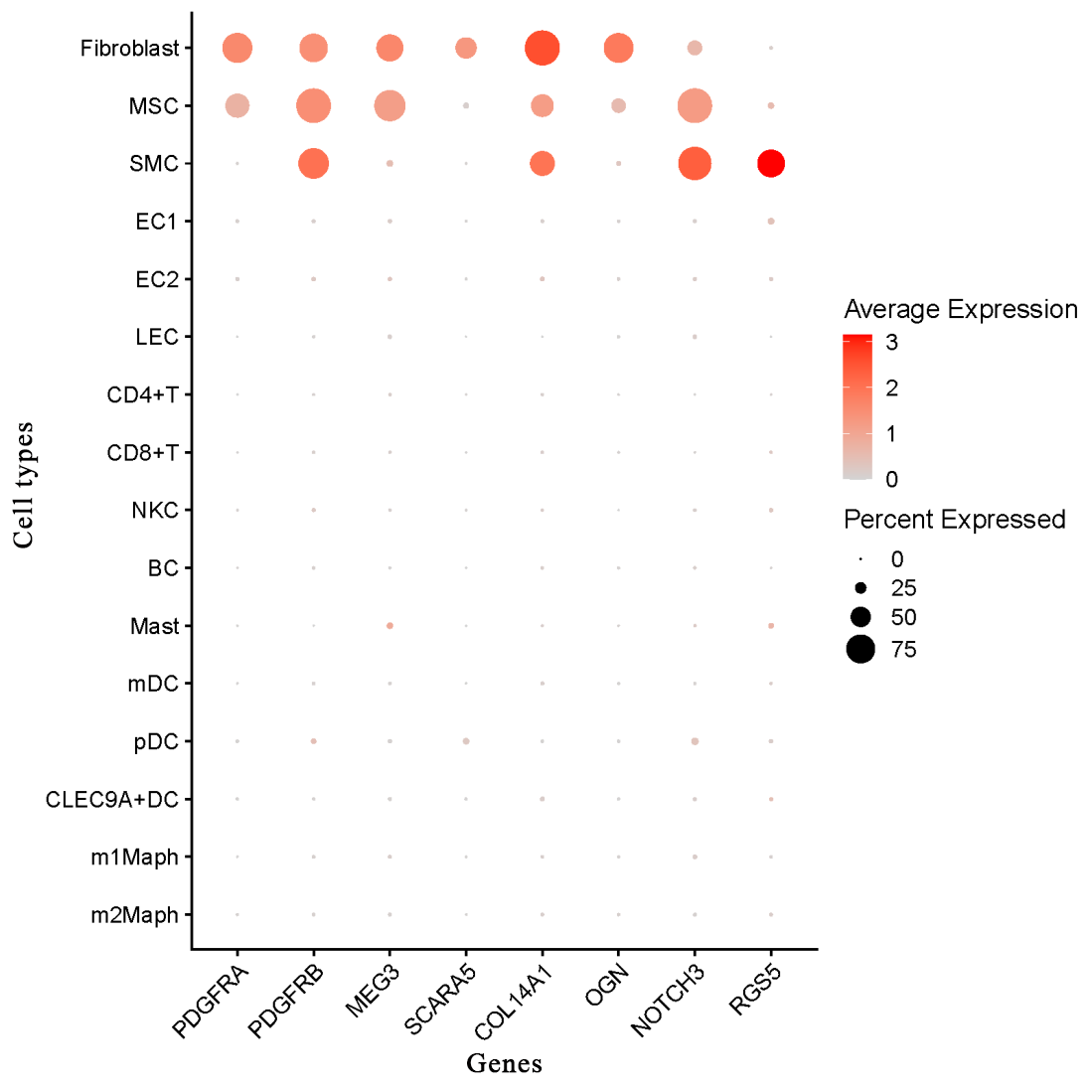


**Fig S5. Expression of** f**ibroblast marker genes**


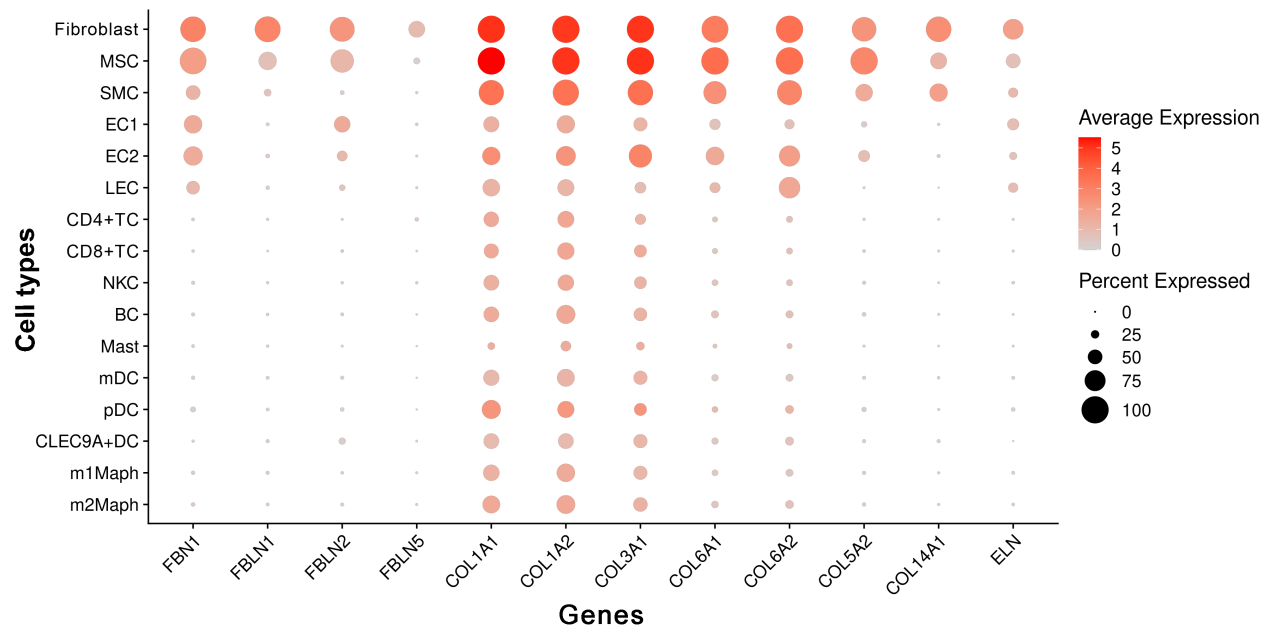
The gene symbols from the left to the right are *PDGFRA*, *PDGFRB*, *MEG3*, *SCARA5*, *COL14A1*, *OGN*, *NOTCH3*, and *RGS5*.
**Fig S6. Expression of** **genes required in extracellular matrix**

The gene symbols from the left to the right are *FBN1*, *FBLN1*, *FBLN2*, *FBLN5*, *COL1A1*, *COL1A2*, *COL3A1*, *COL6A1*, *COL6A2*, *COL5A2*, *COL14A1* and *ELN*.


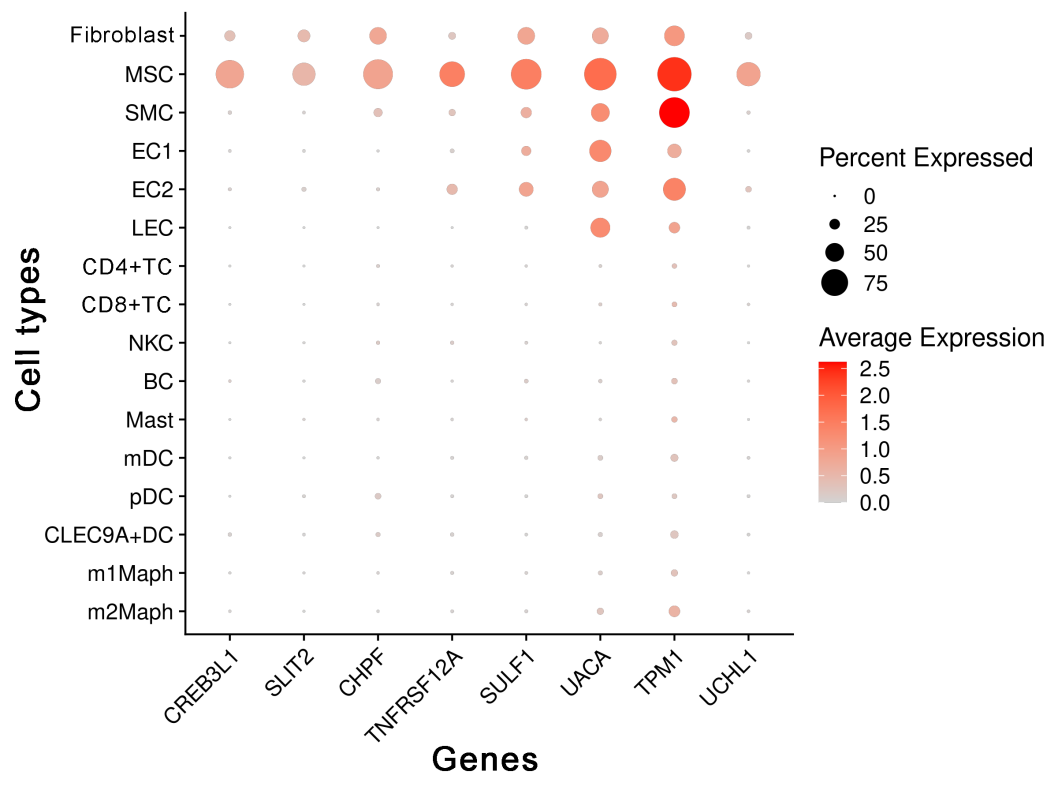


**Fig S7. Expression of** **seven genes in the gene-expression signature of mesenchymal stem cells**

Seven genes (*CREB3L1*, *SLIT2*, *CHPF*, *TNFRSF12A*, *SULF1*, *UACA* and *TPM1*) have been reported to be under-expressed or down-regulated in cancers, whereas the functions of *UCHL1* are still illusive (Detailed later).+


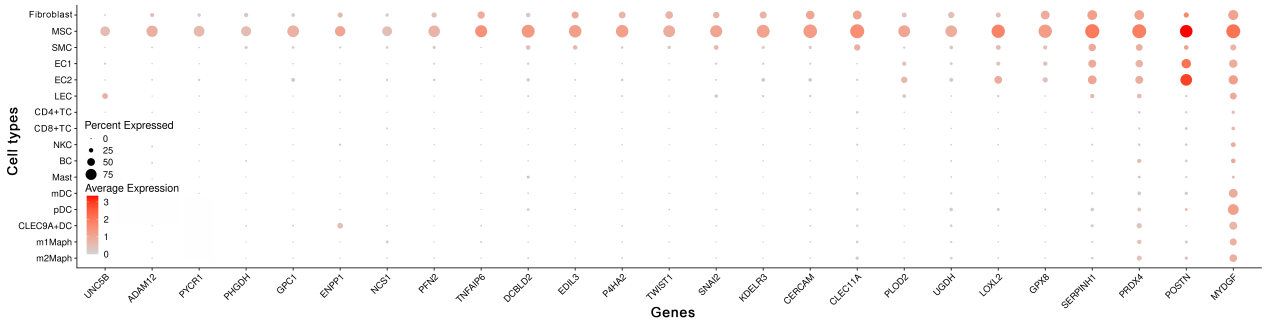


**Fig S8. Expression of** **25 genes in the gene-expression signature of mesenchymal stem cells**

The gene symbols from the left to the right are *UNC5B*, *ADAM12*, *PYCR1*, *PHGDH*, *GPC1, ENPP1, NCS1, PFN2, TNFAIP6, DCBLD2, EDIL3, P4HA2, TWIST1, SNAI2, KDELR3, CERCAM, CLEC11A, PLOD2, UGDH, LOXL2, GPX8, SERPINH1, PRDX4, POSTN* and *MYDGF*.


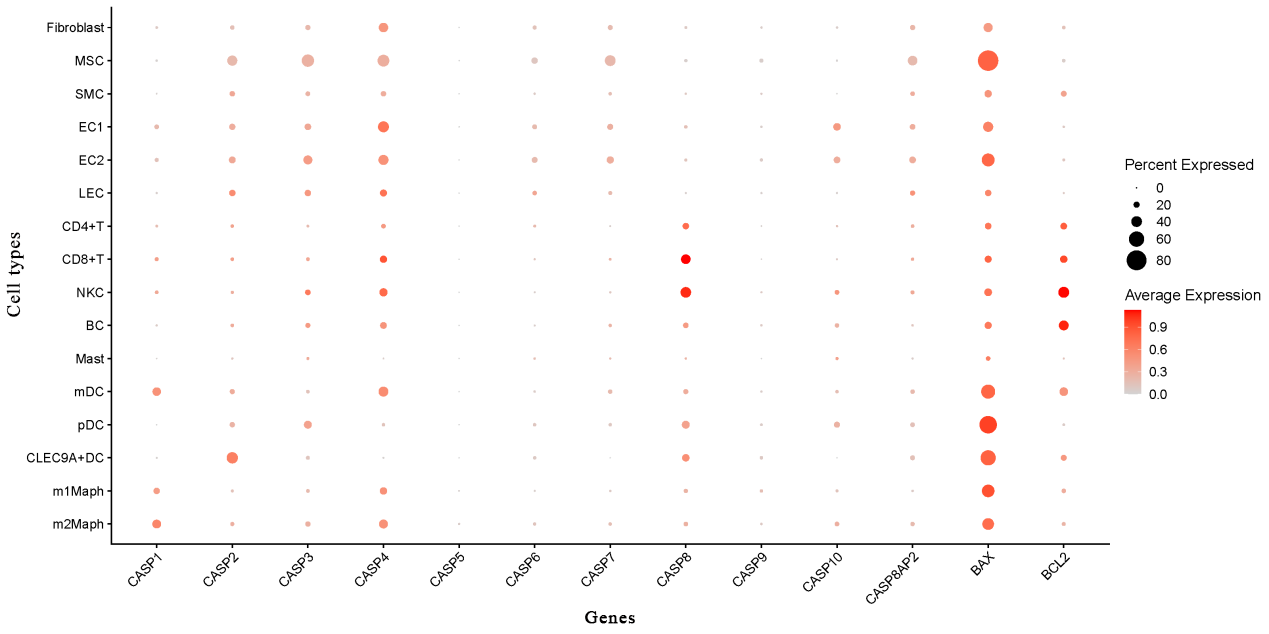


**Fig S9. Expression of marker genes of cell apoptosis in mesenchymal stem cells**

The gene symbols from the left to the right are *CASP1*, *CASP2*, *CASP3*, *CASP4*, *CASP5*, *CASP6*, *CASP7*, *CASP8*, *CASP9*, *CASP10*, *CASP8AP2*, *BAX* and *BCL2*.


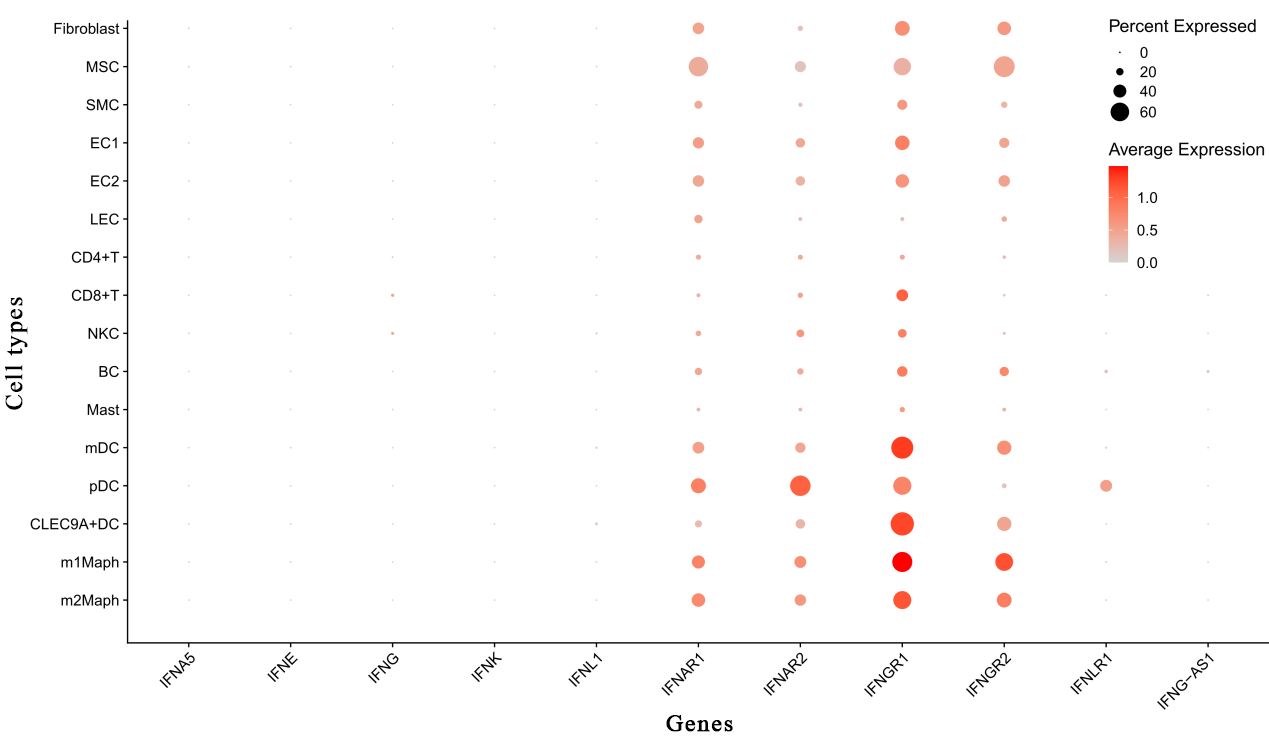


**Fig S10. Expression of genes encoding INFs and their receptors**

The gene symbols from the left to the right are *IFNA5*, *IFNE*, *IFNG*, *IFNK*, *IFNL1*, *IFNAR1*, *IFNAR2*, *IFNGR1*, *IFNGR2*, *IFNLR1* and *IFNG-AS1*.


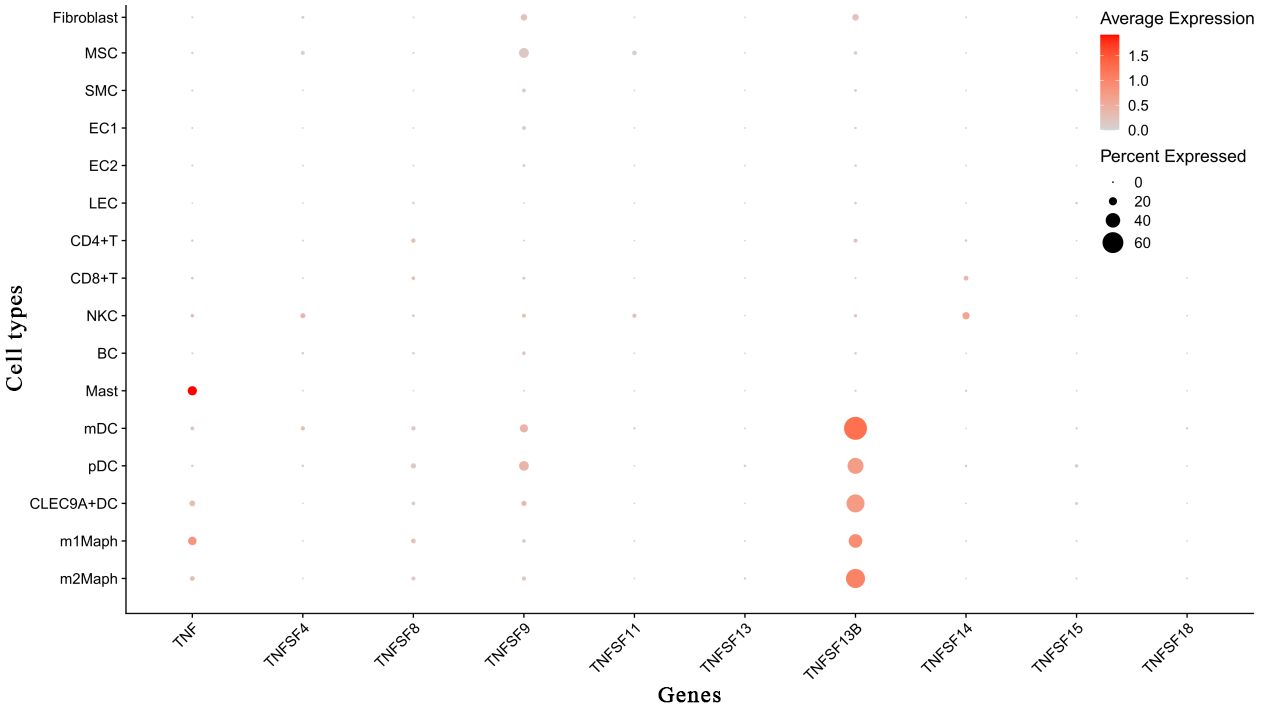


**Fig S11. Expression of genes encoding TNFSFs**

The gene symbols from the left to the right are *TNF*, *TNFSF4*, *TNFSF8*, *TNFSF9*, *TNFSF11*, *TNFSF13*, *TNFSF13B*, *TNFSF14*, *TNFSF15* and *TNFSF18*.

**
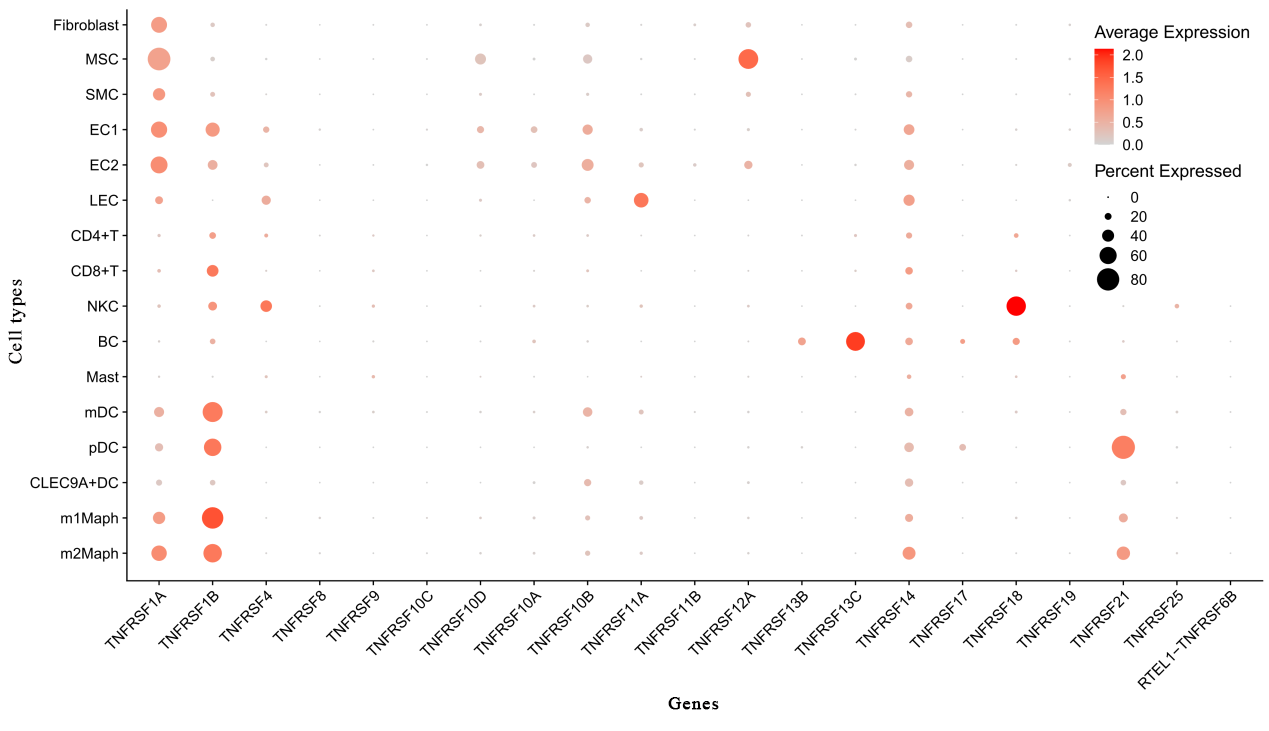
**

**Fig S12. Expression of genes encoding TNFRSFs**

The gene symbols from the left to the right are *TNFRSF1A*, *TNFRSF1B*, *TNFRSF4*, *TNFRSF8*, *TNFRSF9*, *TNFRSF10C*, *TNFRSF10D*, *TNFRSF10A*, *TNFRSF10B*, *TNFRSF11A*, *TNFRSF11B*, *TNFRSF12A*, *TNFRSF13B*, *TNFRSF13C*, *TNFRSF14*, *TNFRSF17*, *TNFRSF18*, *TNFRSF19*, *TNFRSF21*, *TNFRSF25* and *RTEL1-TNFRSF6B*.


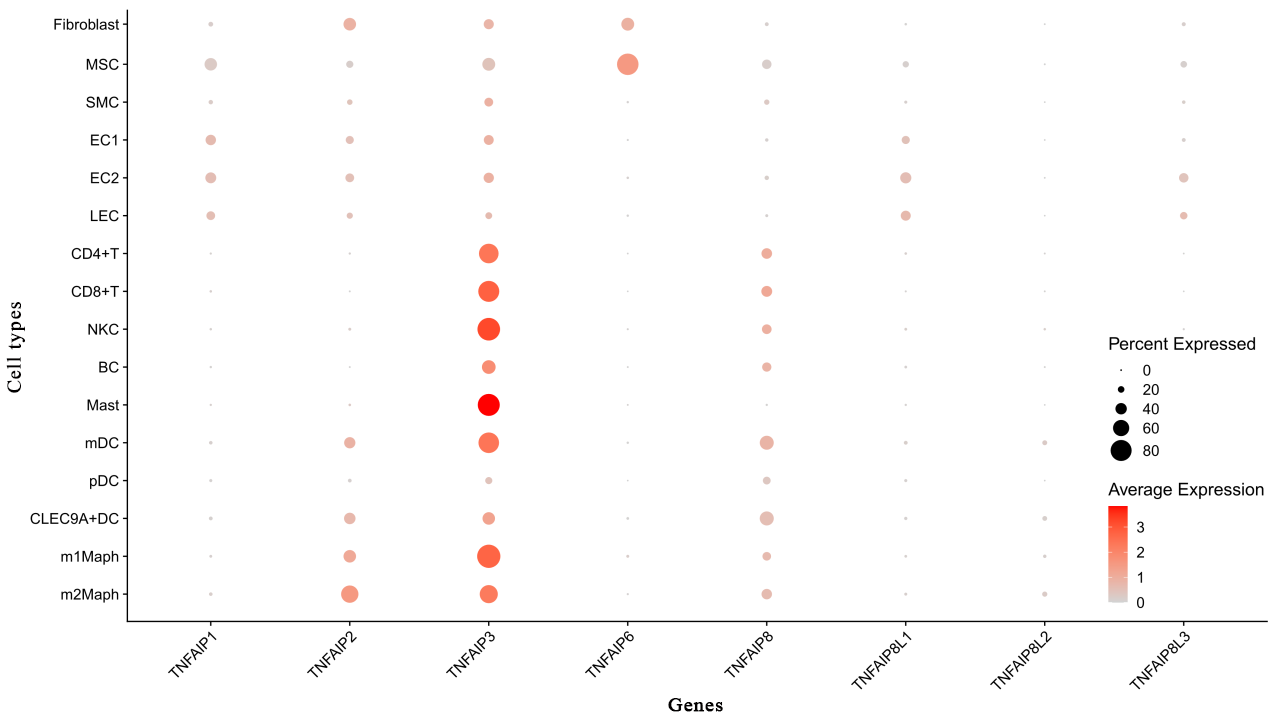


**Fig S13. Expression of genes encoding TNFAIPs**

The gene symbols from the left to the right are *TNFAIP1*, *TNFAIP2*, *TNFAIP3*, *TNFAIP6*, *TNFAIP8*, *TNFAIP8L1*, *TNFAIP8L2* and *TNFAIP8L3*.


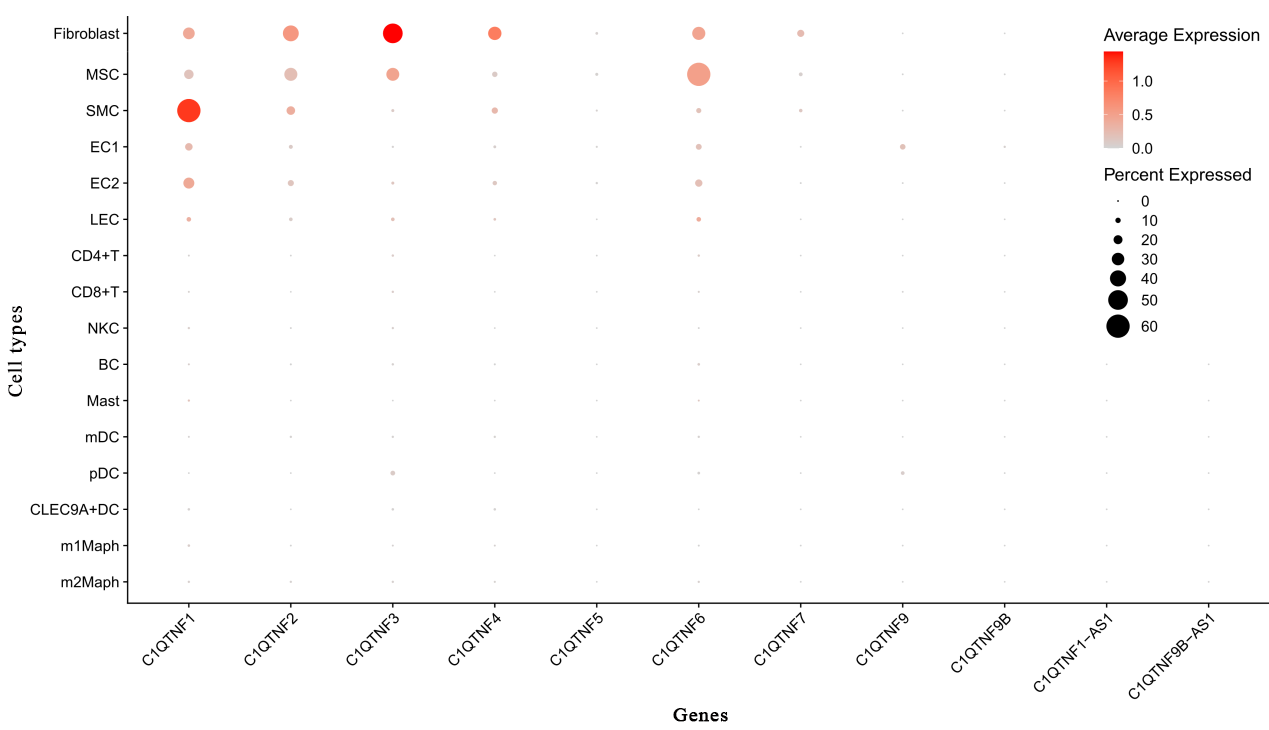


**Fig S14. Expression of genes encoding C1QTNFs**

The gene symbols from the left to the right are *C1QTNF1*, *C1QTNF2*, *C1QTNF3*, *C1QTNF4*, *C1QTNF5*, *C1QTNF6*, *C1QTNF7*, *C1QTNF9*, *C1QTNF9B*, *C1QTNF1-AS1* and *C1QTNF9B-AS1*.


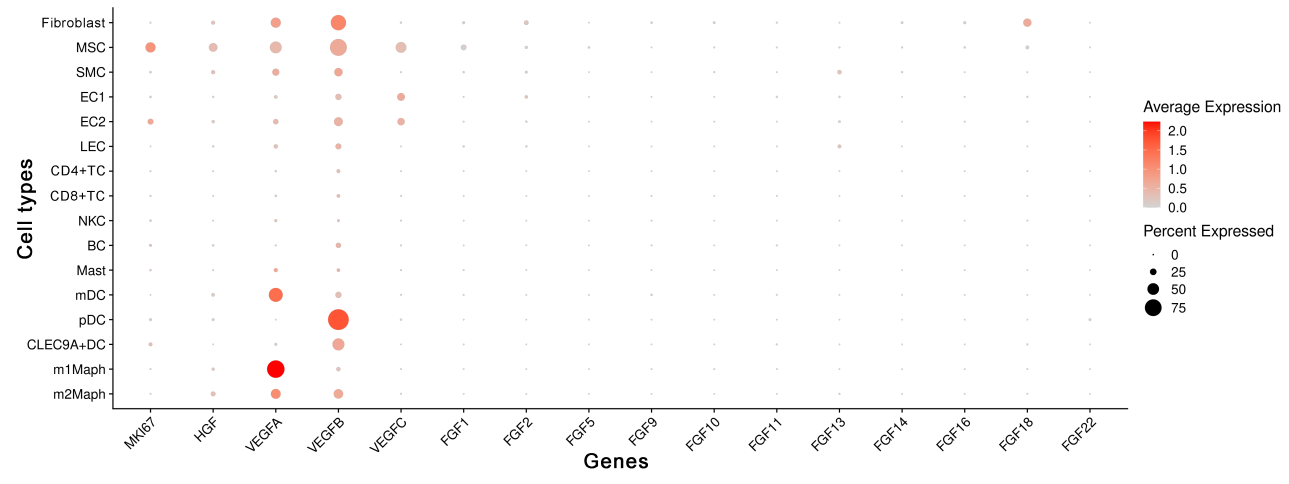


**Fig S15. Expression of VEGF and FGF genes**

The gene symbols from the left to the right are *MKI67*, *HGF*, *VEGFA*, *VEGFB*, *VEGFC*, *FGF1*, *FGF2*, *FGF5*, *FGF9*, *FGF10*, *FGF11*, *FGF13*, *FGF14*, *FGF16*, *FGF18*, and *FGF22*.


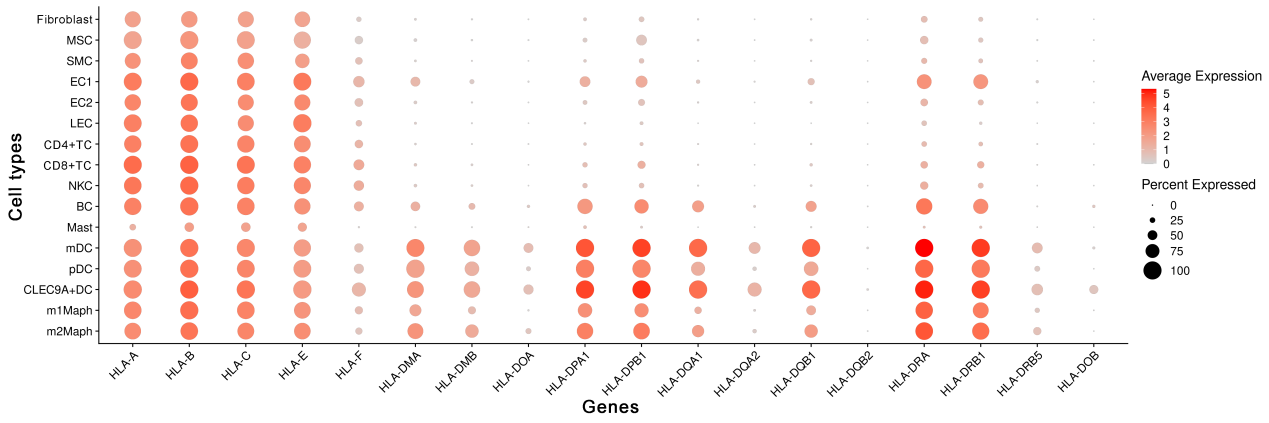


**Fig S16. Expression of** **major histocompatibility complex class II genes**

The gene symbols from the left to the right are *HLA-A*, *HLA-B*, *HLA-C*, *HLA-E*, *HLA-F*, *HLA-DMA*, *HLA-DMB*, *HLA-DOA*, *HLA-DPA1*, *HLA-DPB1*, *HLA-DQA1*, *HLA-DQA2*, *HLA-DQB1*, *HLA-DQB2*, *HLA-DRA*, *HLA-DRB1*, *HLA-DRB5* and *HLA-DOB*.


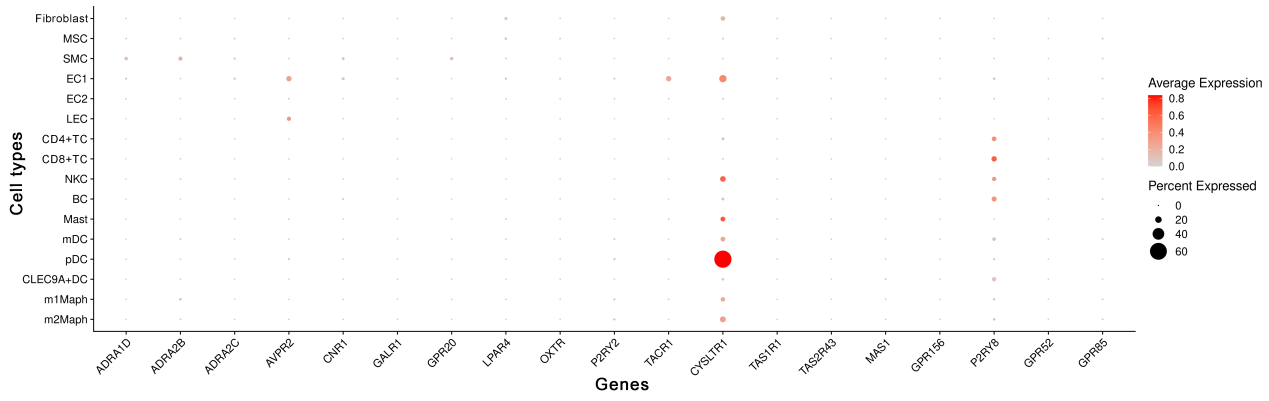


**Fig S17. Expression of** **genes encoding heterotrimeric G protein-coupled receptors**

The gene symbols from the left to the right are *ADRA1D*, *ADRA2B*, *ADRA2C*, *AVPR2*, *CNR1*, *GALR1*,*GPR20*, *LPAR4*, *OXTR*, *P2RY2*, *TACR1*, *CYSLTR1*, *TAS1R1*, *TAS2R43*, *MAS1*, *GPR156*, *P2RY8*, *GPR52* and *GPR85*.


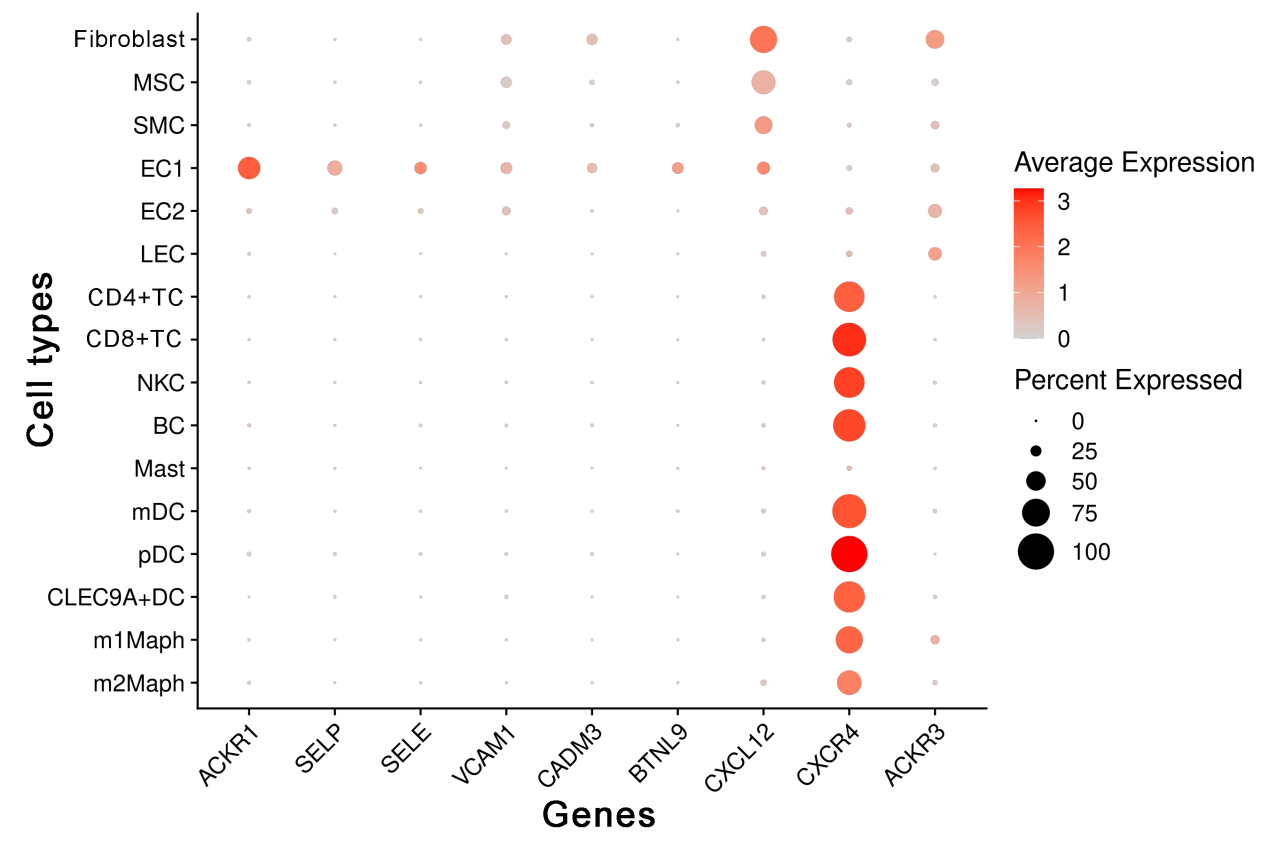


**Fig S18. Expression of** **featured genes of the EC1 cluster**

The gene symbols from the left to the right are *ACKR1*, *SELP*, *SELE*, *VCAM1*, *CADM3*, *BTNL9*, *CXCL12*, *CXCR4* and *ACKR3*.


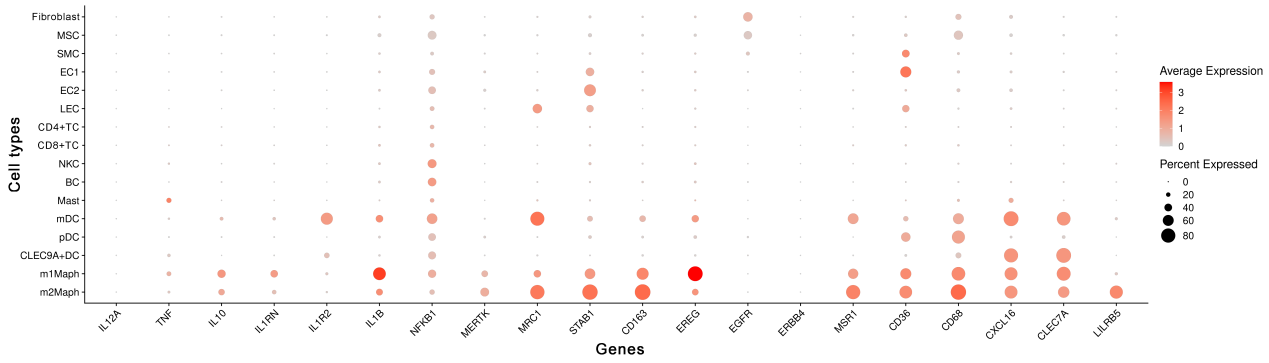


**Fig S19. Expression of** **featured genes of M1 and M2 macrophages**

The gene symbols from the left to the right are *IL12A*, *TNF*, *IL10*, *IL1RN*, *IL1R2*, *IL1B*, *NFKB1*, *MERTK*, *MRC1*, *STAB1*, *CD163*, *EREG*, *EGFR*, *ERBB4*, *MSR1*, *CD36*, *CD68*, *CXCL16*, *CLEC7A* and *LILRB5.*


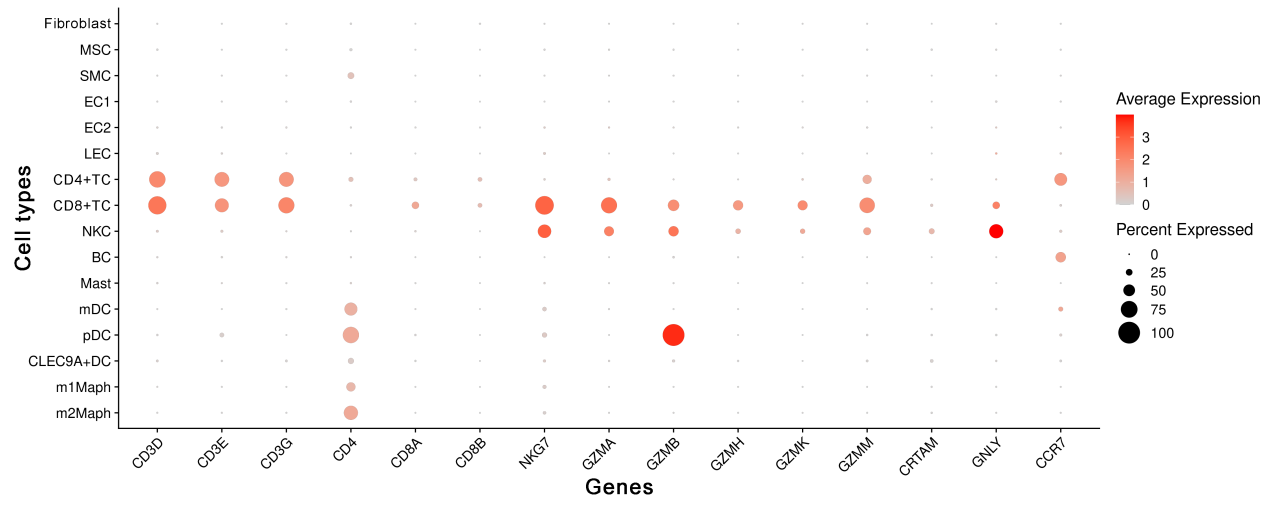


**Fig S20. Expression of** **featured genes to identify CD4+T, CD8+T and NK cells**

The gene symbols from the left to the right are *CD3D*, *CD3E*, *CD3G*, *CD4*, *CD8A*, *CD8B*, *NKG7*, *GZMA*, *GZMB*, *GZMH*, *GZMK*, *GZMM*, *CRTAM*, *GNLY* and *CCR7*.


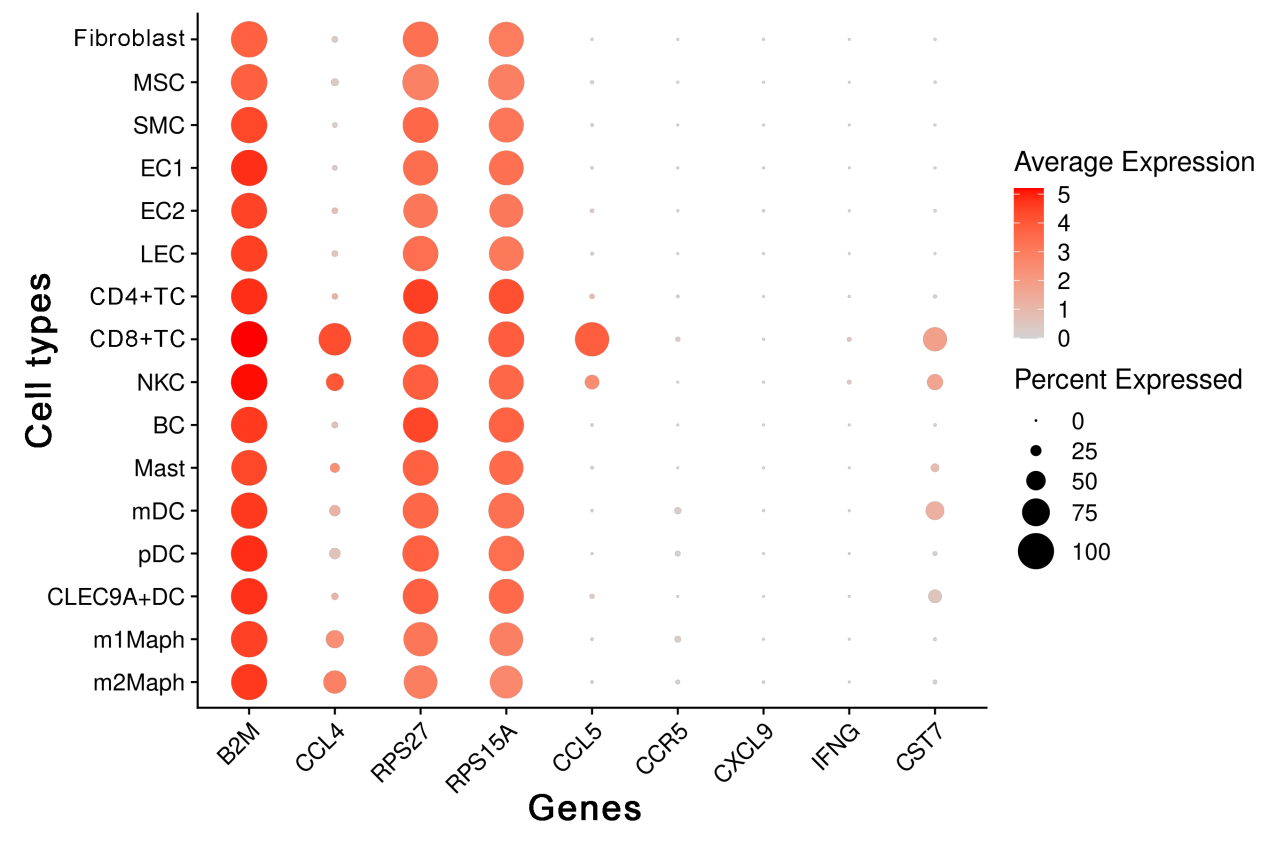


**Fig S21. Expression of** **top highly expressed genes of CD8+T cells**

The gene symbols from the left to the right are *B2M*, *CCL4*, *RPS27*, *RPS15A*, *CCL5*, *CCR5*, *CXCL9*, *INFG* and *CST7*.

## Annotation of featured genes in MSCs

The differential expression analysis between the cells inside and outside MSCs was performed to generate a gene-expression signature, including 63 coding genes and a long noncoding RNA (lncRNA) gene RP11-14N7.2

Further investigation showed that 19 from the 63 coding genes are highly or over-expressed in stem cells. They are *UNC5B, ADAM12, PYCR1, PHGDH, SLIT2, PFN2, TNFAIP6, TNC, EDIL3, TWIST1, SNAI2, PHLDA2, LOXL2, BMP1, COL5A1, POSTN, ID3, COL6A1* and *COL1A1.*

1. ***UNC5B*** was reported as a marker gene of epiblast stem cell;

2. ***ADAM12*** expression is upregulated in claudin-low breast tumors and in cell populations enriched for breast cancer stem cell-like cells [1].

3. ***PYCR1*** is highly expressed in mesenchymal stem cells (MSCs) [2], which originate from epiblast stem cells.

4. ***PHGDH*** expression is required for mitochondrial redox homeostasis, breast cancer stem cell maintenance, and lung metastasis [3].

5. ***SLIT2***, a secreted protein, interacts with its receptor Robo1 to regulate the differentiation of intestinal stem cells and participate in inflammation and tumor development [4].

6. ***PFN2*** is a target of the embryonic stem cell (ESC)-enriched miR-290 family of microRNAs (miRNAs) and an actin/dynamin-binding protein implicated in endocytosis [5].

7. As produced by monocytes/macrophages, mesenchymal stem cells, ***TNFAIP6*** plays an important role in inflammation and tissue remodeling [6].

8. The extracellular matrix protein ***TNC*** is a large glycoprotein expressed in connective tissues and stem cell niches [7].

9. ***EDIL3*** was highly expressed in dermal mesenchymal stem cells (DMSCs) from psoriasis [8].

10. ***TWIST1*** is a basic helix-loop-helix (bHLH) transcription factor which plays essential and pivotal roles in multiple stages of embryonic development and its over-expression induces epithelial-mesenchymal transition (EMT), a key process in the metastases formation of cancer [9].

11. The role of ***SNAI2*** in promoting stem cell function in normal tissues is thought to contribute to cancer stem cell (CSC) behavior [10].

12. ***PHLDA2*** displayed one of the most striking differences in its expression profile in stem versus differentiated cells. It was high in stem cells and virtually undetectable following differentiation, which is also found in mouse trophoblast stem cells [11].

13. The levels of the proteins related to wound healing, including *CTHRC1*, ***LOXL2*** and *LGALS1*, were significantly higher in human amniotic mesenchymal stem cells (hAMSCs-CM) than human amniotic epithelial cells (hAECs-CM) [12].

14. Zhang et al [13] found that high expression of ***BMP1*** promoted osteogenic differentiation of human bone marrow-derived mesenchymal stem cells (BMSCs).

15. Additionally, ***COL5A1*** was more enriched in Ovarian cancer (OC) stem cell-like cells [14].

16. According to a previous study [15], ***POSTN*** is expressed by fibroblasts in the normal tissue and in the stroma of the primary tumor and plays a role in cancer stem cell maintenance and metastasis.

17. ***ID3*** has been reported to promote cancer stem cells [16].

18. Previous microarray studies showed that the expression levels of ***COL6A1*** and ***COL6A2*** in bulge stem cells are higher than in differentiated keratinocytes, suggesting that collagen VI may contribute to bulge function and hair development [17].

19. However, both ***COL1A1*** and ***COL3A1*** gene expression could be detected in non-stretched mesenchymal stem cells [18].

Further investigation showed that at least 33 from the 63 coding are now being studied in cancers. Among them, 25 genes (*UNC5B, ADAM12, PYCR1, PHGDH, GPC1, ENPP1, NCS1, PFN2, TNFAIP6, DCBLD2, EDIL3, P4HA2, TWIST1, SNAI2, KDELR3, CERCAM, CLEC11A, PLOD2, UGDH, LOXL2, GPX8, SERPINH1, PRDX4, POSTN* and *MYDGF*) are over-expressed or up-regulated in cancers, whereas seven genes (*CREB3L1, SLIT2, CHPF, TNFRSF12A, SULF1, UACA* and *TPM1*) are low expressed or down-regulated. In addition, *UCHL1* acts as a tumor promoter in pancreatic cancer, prostate cancer and lung cancer. However, it acts as a tumor suppressor in ovarian cancer, hepatocellular cancer and nasopharyngeal carcinoma.

**1**. Up-regulated expression of ***netrin-1*** and ***UNC5B*** has been observed in breast cancer patients with distant metastasis [19].

**2**. ***ADAM12*** is expressed at low levels in most normal adult tissues, but it is expressed at higher levels in a large proportion of human carcinomas, including breast, gastric, colon carcinomas, and liver metastases [20].

**3**. ***PYCR1*** encodes an enzyme involved in cell metabolism and knockdown of PYCR1 inhibits cell proliferation. ***PYCR1*** is highly expressed in gastric cancer (GC) and acts as a mitochondrial oncogene to induce cancer progression by enhancing tumor proliferation and responding to metabolic stress [21].

**4**. ***PHGDH*** encodes an enzyme of serine biosynthesis over-expressed in various types of cancer. and a new series of PHGDH inhibitors selectively block proliferation of PHGDH-dependent cancer cells [22].

**5**. ***GPC1*** is a membrane anchored protein reported previously as over-expressed in a variety of cancers, including breast and pancreas cancer [23].

**6**. ***ENPP1*** encodes a critical phosphodiesterase that promotes metastasis by selectively degrading extracellular cyclic GMP-AMP (cGAMP). In human cancers, the expression level of ***ENPP1*** correlates with reduced immune cell infiltration and resistance to anti-PD-1/PD-L1 treatment [24]. Further, in clinical specimens, levels of ***ENPP1*** were significantly elevated in human primary breast tumors relative to normal mammary epithelium, with highest levels observed in breast-bone metastasis as determined by qRT-PCR and immunohistochemical analysis [25].

**7**. ***NCS1***, a calcium-binding protein, is associated with clinicopathologic features of aggressiveness in breast cancer cells and worse outcome in two breast cancer patient [26].

**8**. ***PFN2*** expression is up-regulated in breast cancer tissue, and the upregulation of ***PFN2*** is associated with worse prognosis [27].

**9**. ***TNFAIP6*** is also a biomarker in colorectal cancer patients who have increased expression in peripheral blood cells relative to controls [28].

**10**. ***DCBLD2*** is up-regulated in clinical specimens of glioblastomas and head and neck cancers (HNCs) and is required for EGFR-stimulated tumorigenesis [29].

**11**. ***EDIL3*** expression levels were increased in paclitaxel-resistant breast and prostate cancer cells, and in subsets of high-grade breast and prostate tumors [30].

**12**. ***P4HA2*** is up-regulated in cervical cancer and P4HA2 overexpression correlates with poor prognosis of cervical cancer patients[31].

**13**. ***TWIST1*** is over-expressed in malignant tumors, such as breast cancer, esophageal squamous carcinoma, thyroid cancer, lung cancer, gastric carcinoma, colorectal cancer, hepatocellular carcinoma, pancreatic cancer, cervical carcinoma and it usually associated with poor prognosis [32].

**14**. ***SNAI2*** is highly expressed in Rhabdomyosarcoma (RMS) and is regulated by MYOD bound super enhancers [33].

**15**. ***KDELR3*** regulates the metastasis suppressor and the melanoblast transcriptome can be mined to uncover targetable pathways for melanoma therapy [34].

**16**. In conclusion, cell adhesion molecule ***CERCAM*** is over-expressed in bladder cancer tissues [35].

**17**. ***CLEC11A*** is also associated with the development of several cancers, including leukemia, multiple myeloma, and gastrointestinal tract tumors [36].

**18**. Up-regulation of ***PLOD2*** has been observed in various human malignancies, including breast cancer, biliary cancer, colorectal cancer, glioma, and liver cancer, as well as in CESC [37].

**19**. Subsequently, ***UGDH*** was found elevated in epithelial-mesenchymal transition of invasive breast cancer, where its role is hypothesized to be in support of metabolic reprogramming that fuels HA production [38].

**20**. Over-expression of ***LOXL2*** and *SERPINH1* was observed in clinical specimens of lung cancer and fibrotic lesions [39].

**21**. Gastric cancer (GC) patients exhibit increased ***GPX8*** expression [40].

**22**. Over-expression of *LOXL2* and ***SERPINH1*** was observed in clinical specimens of lung cancer and fibrotic lesions [39].

**23**. Recent studies revealed the over-expression of ***PRDX4*** in several kinds of cancers, such as breast cancer, prostate cancer, ovarian cancer, colorectal cancer, and lung cancer [41].

**24**. ***POSTN*** is also a protein involved in the processes of neoplastic transformation, and high levels have been found in various types of cancer in humans, including cancer of the breast, ovary, lungs, prostate, kidneys, intestine and pancreas [42].

**25**. ***MYDGF*** was found over-expressed in approximately two-thirds of Hepatocellular Carcinoma (HCC) tissues [43].

**26.** Immunohistochemical evaluation showed that **CREB3L1** protein expression is decreased in bladder cancer tissues [44].

**27**. Low expression of ***SLIT2*** correlates with poor prognosis and promotes metastasis in esophageal squamous cell carcinoma (ESCC) [45].

**28**. ***CHPF*** was frequently down-regulated in hepatocellular carcinoma (HCC) tumors compared with adjacent non-tumor tissues [46].

**29**. ***TNFRSF12A*** expression may be a potential useful prognostic molecular biomarker of bad survival in thyroid cancer. Decreased expression of ***TNFRSF12A*** in thyroid cancer was associated with advanced clinical pathologic factors [47].

**30**. ***SULF1*** expression is down-regulated in gastric cancer [48].

**31**. The expression of *APIP* and ***UACA*** genes is down-regulated on the level of both mRNA and protein in non-small cell lung carcinoma (NSCLC) cells and tumours [49].

**32**. It was reported that ***TPM1*** expression was down-regulated in multiple cancers types, including renal, breast, esophageal, colorectal and ovarian cancer [50].

**References**

1. S. Duhachek-Muggy, Y. Qi, R. Wise, L. Alyahya, H. Li, J. Hodge and A. Zolkiewska, *Metalloprotease-disintegrin ADAM12 actively promotes the stem cell-like phenotype in claudin-low breast cancer.* Mol Cancer, 2017. **16**(1): p. 32.

2. G. Tejedor, R. Contreras-Lopez, A. Barthelaix, M. Ruiz and F. Djouad, *Pyrroline-5-Carboxylate Reductase 1 Directs the Cartilage Protective and Regenerative Potential of Murphy Roths Large Mouse Mesenchymal Stem Cells.* Frontiers in Cell and Developmental Biology, 2021. **9**: p. 604756.

3. D. Samanta, Y. Park, S.A. Andrabi, L.M. Shelton, D.M. Gilkes and G.L. Semenza, *PHGDH Expression Is Required for Mitochondrial Redox Homeostasis, Breast Cancer Stem Cell Maintenance, and Lung Metastasis.* Cancer Res, 2016. **76**(15): p. 4430-42.

4. J. Xie, L. Li, S. Deng, J. Chen, Q. Gu, H. Su, L. Wen, S. Wang, C. Lin, C. Qi, Q. Zhang, J. Li, X. He, W. Li, L. Wang and L. Zheng, *Slit2/Robo1 Mitigates DSS-induced Ulcerative Colitis by Activating Autophagy in Intestinal Stem Cell.* Int J Biol Sci, 2020. **16**(11): p. 1876-1887.

5. C. Sangokoya and R. Blelloch, *MicroRNA-dependent inhibition of PFN2 orchestrates ERK activation and pluripotent state transitions by regulating endocytosis.* Proc Natl Acad Sci U S A, 2020. **117**(34): p. 20625-20635.

6. X. Zhang, J. Xue, H. Yang, T. Zhou and G. Zu, *TNFAIP6 promotes invasion and metastasis of gastric cancer and indicates poor prognosis of patients.* Tissue Cell, 2021. **68**: p. 101455.

7. C.M. Lowy and T. Oskarsson, *Tenascin C in metastasis: A view from the invasive front.* Cell Adh Migr, 2015. **9**(1-2): p. 112-24.

8. X. Niu, Q. Han, Y. Liu, J. Li, R. Hou, J. Li and K. Zhang, *Psoriasis-associated angiogenesis is mediated by EDIL3.* Microvasc Res, 2020. **132**: p. 104056.

9. Q.Q. Zhu, C. Ma, Q. Wang, Y. Song and T. Lv, *The role of TWIST1 in epithelial-mesenchymal transition and cancers.* Tumor Biology, 2016.

10. W. Zhou, K.M. Gross and C. Kuperwasser, *Molecular regulation of Snai2 in development and disease.* J Cell Sci, 2019. **132**(23).

11. L.N. Kent, T. Konno and M.J. Soares, *Phosphatidylinositol 3 kinase modulation of trophoblast cell differentiation.* BMC Dev Biol, 2010. **10**: p. 97.

12. D. He, F. Zhao, H. Jiang, Y. Kang, Y. Song, X. Lin, P. Shi, T. Zhang and X. Pang, *LOXL2 from human amniotic mesenchymal stem cells accelerates wound epithelialization by promoting differentiation and migration of keratinocytes.* Aging (Albany NY), 2020. **12**(13): p. 12960-12986.

13. Y. Zhang, B. Chen, D. Li, X. Zhou and Z. Chen, *LncRNA NEAT1/miR-29b-3p/BMP1 axis promotes osteogenic differentiation in human bone marrow-derived mesenchymal stem cells.* Pathol Res Pract, 2019. **215**(3): p. 525-531.

14. J. Zhang, J. Zhang, F. Wang, X. Xu, X. Li, W. Guan, T. Men and G. Xu, *Overexpressed COL5A1 is correlated with tumor progression, paclitaxel resistance, and tumor-infiltrating immune cells in ovarian cancer.* J Cell Physiol, 2021.

15. I. Malanchi, A. Santamaria-Martínez, E. Susanto, H. Peng, H.-A. Lehr, J.-F. Delaloye and J. Huelsken, *Interactions between cancer stem cells and their niche govern metastatic colonization.* Nature, 2012. **481**(7379): p. 85-89.

16. L. Huang, J. Cai, H. Guo, J. Gu, Y. Tong, B. Qiu, C. Wang, M. Li, L. Xia, J. Zhang, H. Wu, X. Kong and Q. Xia, *ID3 Promotes Stem Cell Features and Predicts Chemotherapeutic Response of Intrahepatic Cholangiocarcinoma.* Hepatology, 2019. **69**(5): p. 1995-2012.

17. P. Chen, M. Cescon and P. Bonaldo, *Lack of Collagen VI Promotes Wound-Induced Hair Growth.* J Invest Dermatol, 2015. **135**(10): p. 2358-2367.

18. C.H. Ku, P.H. Johnson, P. Batten, P. Sarathchandra, R.C. Chambers, P.M. Taylor, M.H. Yacoub and A.H. Chester, *Collagen synthesis by mesenchymal stem cells and aortic valve interstitial cells in response to mechanical stretch.* Cardiovasc Res, 2006. **71**(3): p. 548-56.

19. C. Kong, Z. Bo, C. Piao, Z. Zhe, Y. Zhu and Q. Li, *Overexpression of UNC5B in bladder cancer cells inhibits proliferation and reduces the volume of transplantation tumors in nude mice.* BMC Cancer, 2016. **16**(1): p. 892.

20. M. Kveiborg, C. Fröhlich, R. Albrechtsen, V. Tischler, N. Dietrich, P. Holck, P. Kronqvist, F. Rank, A.M. Mercurio and U.M. Wewer, *A role for ADAM12 in breast tumor progression and stromal cell apoptosis.* Cancer Res, 2005. **65**(11): p. 4754-61.

21. S. Xiao, S. Li, Z. Yuan and L. Zhou, *Pyrroline-5-carboxylate reductase 1 (PYCR1) upregulation contributes to gastric cancer progression and indicates poor survival outcome.* Annals of Translational Medicine, 2020. **8**(15): p. 937-937.

22. C. Frezza, *Addicted to serine.* Nature Chemical Biology, 2016. **12**(6): p. 389-390.

23. S.A. Melo, L.B. Luecke, C. Kahlert, A.F. Fernandez, S.T. Gammon, J. Kaye, V.S. LeBleu, E.A. Mittendorf, J. Weitz, N. Rahbari, C. Reissfelder, C. Pilarsky, M.F. Fraga, D. Piwnica-Worms and R. Kalluri, *Glypican-1 identifies cancer exosomes and detects early pancreatic cancer.* Nature, 2015. **523**(7559): p. 177-82.

24. J. Li, M.A. Duran, N. Dhanota, W.K. Chatila and S.F. Bakhoum, *Metastasis and Immune Evasion from Extracellular cGAMP Hydrolysis.* Cancer Discovery, 2020: p. CD-20-0387.

25. W.M. Lau, M. Doucet, R. Stadel, D. Huang, K.L. Weber and S.L. Kominsky, *Enpp1: a potential facilitator of breast cancer bone metastasis.* PLoS One, 2013. **8**(7): p. e66752.

26. J.E. Apasu, D. Schuette, R. LaRanger, J.A. Steinle, L.D. Nguyen, H.K. Grosshans, M. Zhang, W.L. Cai, Q. Yan, M.E. Robert, M. Mak and B.E. Ehrlich, *Neuronal calcium sensor 1 (NCS1) promotes motility and metastatic spread of breast cancer cells in vitro and in vivo.* Faseb j, 2019. **33**(4): p. 4802-4813.

27. Y. Ling, Q. Cao, Y. Liu, J. Zhao, Y. Zhao, K. Li, Z. Chen, X. Du, X. Huo, H. Kang and Z. Chen, *Profilin 2 (PFN2) promotes the proliferation, migration, invasion and epithelial-to-mesenchymal transition of triple negative breast cancer cells.* Breast Cancer, 2021. **28**(2): p. 368-378.

28. J.M. Evans, H.G. Parker, G.R. Rutteman, J. Plassais, G.C.M. Grinwis, A.C. Harris, S.E. Lana and E.A. Ostrander, *Multi-omics approach identifies germline regulatory variants associated with hematopoietic malignancies in retriever dog breeds.* PLoS Genet, 2021. **17**(5): p. e1009543.

29. H. Feng, G.Y. Lopez, C.K. Kim, A. Alvarez, C.G. Duncan, R. Nishikawa, M. Nagane, A.J. Su, P.E. Auron, M.L. Hedberg, L. Wang, J.J. Raizer, J.A. Kessler, A.T. Parsa, W.Q. Gao, S.H. Kim, M. Minata, I. Nakano, J.R. Grandis, R.E. McLendon, D.D. Bigner, H.K. Lin, F.B. Furnari, W.K. Cavenee, B. Hu, H. Yan and S.Y. Cheng, *EGFR phosphorylation of DCBLD2 recruits TRAF6 and stimulates AKT-promoted tumorigenesis.* J Clin Invest, 2014. **124**(9): p. 3741-56.

30. J. Gasca, M.L. Flores, R. Jiménez-Guerrero, M.E. Sáez, I. Barragán, M. Ruíz-Borrego, M. Tortolero, F. Romero, C. Sáez and M.A. Japón, *EDIL3 promotes epithelial-mesenchymal transition and paclitaxel resistance through its interaction with integrin α(V)β(3) in cancer cells.* Cell Death Discov, 2020. **6**: p. 86.

31. Y. Cao, Q. Han, J. Li, Y. Jia, R. Zhang and H. Shi, *P4HA2 contributes to cervical cancer progression via inducing epithelial-mesenchymal transition.* J Cancer, 2020. **11**(10): p. 2788-2799.

32. H. Ren, P. Du, Z. Ge, Y. Jin, D. Ding, X. Liu and Q. Zou, *TWIST1 and BMI1 in Cancer Metastasis and Chemoresistance.* J Cancer, 2016. **7**(9): p. 1074-80.

33. S. Pomella, P. Sreenivas, B.E. Gryder, L. Wang, D. Milewski, M. Cassandri, K. Baxi, N.R. Hensch, E. Carcarino, Y. Song, H.C. Chou, M.E. Yohe, B.Z. Stanton, B. Amadio, I. Caruana, C. De Stefanis, R. De Vito, F. Locatelli, Y. Chen, E.Y. Chen, P. Houghton, J. Khan, R. Rota and M.S. Ignatius, *Interaction between SNAI2 and MYOD enhances oncogenesis and suppresses differentiation in Fusion Negative Rhabdomyosarcoma.* Nat Commun, 2021. **12**(1): p. 192.

34. K.L. Marie, A. Sassano, H.H. Yang, A.M. Michalowski, H.T. Michael, T. Guo, Y.C. Tsai, A.M. Weissman, M.P. Lee, L.M. Jenkins, M.R. Zaidi, E. Pérez-Guijarro, C.P. Day, K. Ylaya, S.M. Hewitt, N.L. Patel, H. Arnheiter, S. Davis, P.S. Meltzer, G. Merlino and P.J. Mishra, *Melanoblast transcriptome analysis reveals pathways promoting melanoma metastasis.* Nat Commun, 2020. **11**(1): p. 333.

35. Y. Zuo, X. Xu, M. Chen and L. Qi, *The oncogenic role of the cerebral endothelial cell adhesion molecule (CERCAM) in bladder cancer cells in vitro and in vivo.* Cancer Med, 2021. **10**(13): p. 4437-4450.

36. M. Wang, J. Guo, L. Zhang, V. Kuek, J. Xu and J. Zou, *Molecular structure, expression, and functional role of Clec11a in skeletal biology and cancers.* J Cell Physiol, 2020. **235**(10): p. 6357-6365.

37. G. Li, X. Wang and G. Liu, *PLOD2 Is a Potent Prognostic Marker and Associates with Immune Infiltration in Cervical Cancer.* Biomed Res Int, 2021. **2021**: p. 5512340.

38. B.M. Zimmer, J.J. Barycki and M.A. Simpson, *Integration of Sugar Metabolism and Proteoglycan Synthesis by UDP-glucose Dehydrogenase.* J Histochem Cytochem, 2021. **69**(1): p. 13-23.

39. K. Kamikawaji, N. Seki, M. Watanabe, H. Mataki, T. Kumamoto, K. Takagi, K. Mizuno and H. Inoue, *Regulation of LOXL2 and SERPINH1 by antitumor microRNA-29a in lung cancer with idiopathic pulmonary fibrosis.* J Hum Genet, 2016. **61**(12): p. 985-993.

40. X. Zhang, D. Zhan, Y. Li, H. Wang, C. Cheng, Y. Yao and J. Jia, *Glutathione Peroxidase 8 as a Prognostic Biomarker of Gastric Cancer: An Analysis of The Cancer Genome Atlas (TCGA) Data.* Med Sci Monit, 2020. **26**: p. e921775.

41. W. Jia, P. Chen and Y. Cheng, *PRDX4 and Its Roles in Various Cancers.* Technol Cancer Res Treat, 2019. **18**: p. 1533033819864313.

42. P. Borecka, K. Ratajczak-Wielgomas, R. Ciaputa, M. Kandefer-Gola and M. Nowak, *Expression of Periostin in Cancer-associated Fibroblasts in Mammary Cancer in Female Dogs.* In vivo (Athens, Greece), 2020. **34**(3): p. 1017-1026.

43. H. Sunagozaka, M. Honda, T. Yamashita, R. Nishino, H. Takatori, K. Arai, T. Yamashita, Y. Sakai and S. Kaneko, *Identification of a secretory protein c19orf10 activated in hepatocellular carcinoma.* Int J Cancer, 2011. **129**(7): p. 1576-85.

44. M. Rose, C. Schubert, L. Dierichs, N.T. Gaisa, M. Heer, A. Heidenreich, R. Knüchel and E. Dahl, *OASIS/CREB3L1 is epigenetically silenced in human bladder cancer facilitating tumor cell spreading and migration in vitro.* Epigenetics, 2014. **9**(12): p. 1626-40.

45. R.C. Tseng, J.M. Chang, J.H. Chen, W.R. Huang, Y.A. Tang, I.Y. Kuo, J.J. Yan, W.W. Lai and Y.C. Wang, *Deregulation of SLIT2-mediated Cdc42 activity is associated with esophageal cancer metastasis and poor prognosis.* J Thorac Oncol, 2015. **10**(1): p. 189-98.

46. C.H. Liu, B.R. Wu, Y.J. Ho, Y.H. Chu and W.C. Liao, *CHPF Regulates the Aggressive Phenotypes of Hepatocellular Carcinoma Cells via the Modulation of the Decorin and TGF-β Pathways.* Cancers, 2021. **13**(6): p. 1261.

47. Z.H. Wu, X. Niu, G.H. Wu and Q. Cheng, *Decreased expression of TNFRSF12A in thyroid gland cancer predicts poor prognosis: A study based on TCGA data.* Medicine (Baltimore), 2020. **99**(34): p. e21882.

48. S. Junnila, A. Kokkola, T. Mizuguchi, K. Hirata, M.L. Karjalainen-Lindsberg, P. Puolakkainen and O. Monni, *Gene expression analysis identifies over-expression of CXCL1, SPARC, SPP1, and SULF1 in gastric cancer.* Genes Chromosomes Cancer, 2010. **49**(1): p. 28-39.

49. E. Moravcikova, E. Krepela, J. Prochazka, I. Rousalova, J. Cermak and K. Benkova, *Down-regulated expression of apoptosis-associated genes APIP and UACA in non-small cell lung carcinoma.* Int J Oncol, 2012. **40**(6): p. 2111-21.

50. H. Pan, L. Gu, B. Liu, Y. Li, Y. Wang, X. Bai, L. Li, B. Wang, Q. Peng, Z. Yao and Z. Tang, *Tropomyosin-1 acts as a potential tumor suppressor in human oral squamous cell carcinoma.* PLoS One, 2017. **12**(2): p. e0168900.
