# Supplementary material for: Single-Cell Transcriptome Analysis Reveals Mesenchymal Stem Cells in Cavernous Hemangioma
Source: Front Cell Dev Biol. 2022 Jul 5;10:916045. doi: 10.3389/fcell.2022.916045 (PMC9294370; doi:10.3389/fcell.2022.916045)
Supplement: Supplementary file 2 [file DataSheet1.ZIP › s1.docx]

# Supplementary 1

## Patient information

The patient is a 6-year old child (Admission number: 1107075) without past medical and familiar history. A lesion (deep dermal, blue hue) in the hypothenar region of his right hand was estimated as a diameter of 1.5 cm by observation (**Figure S1**). This lesion was present at birth and grew concomitantly with the patient with a consistent color tone. The patient had his right hand wounded on November 4^th^, 2020, and then was admitted to department of paediatric surgery, Tianjin Medical University General Hospital, on November 7^th^, 2020.


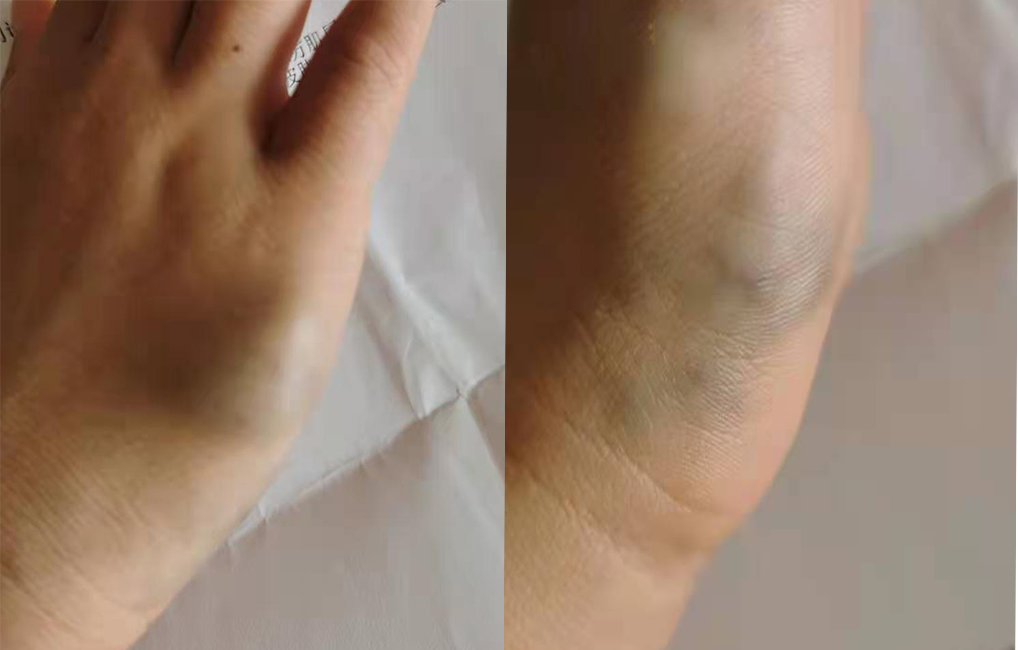


**Figure S1. The tumor in the hypothenar region.**

Doppler ultrasound revealed that the hypoechoic area of the muscle layer in the tumor is about 1.5×0.5 cm and the deepest position in the hypoechoic area of the subcutaneous fat layer is about 0.4 cm.

The results of Magnetic resonance imaging (MRI) confirmed multiple nodules in the subcutaneous soft tissue in the hypothenar region of the right hand by T1- weighted images (**Figure S2**).


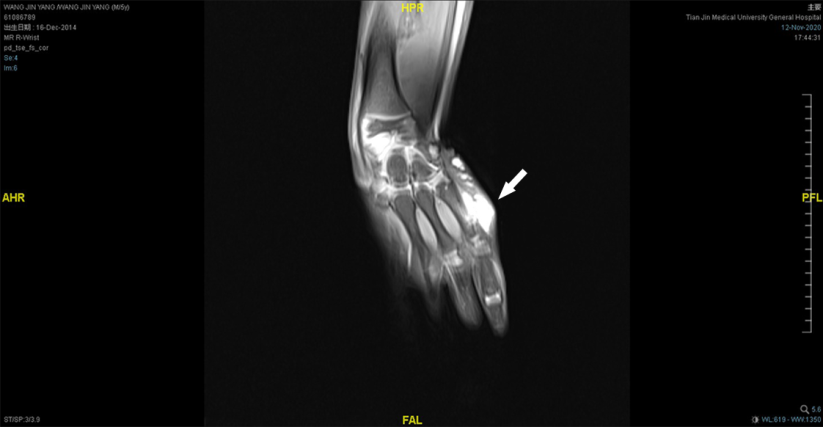


**Figure S2. The results of Magnetic resonance imaging.** The tumor was indicated by an arrow.

## Inspection report

Table S1. Inspection report

| **Indication** | **Result** | **Normal Range** | **Unit** |
| --- | --- | --- | --- |
| WBC# | 11.81 | 4.00-10.00 | *10^9^/L |
| RBC# | 4.86 | 4.00-4.50 | *10**^12^**/L |
| HGB# | 141 | 120-160 | g/L |
| PLT# | 320 | 100-300 | *10**^9^**/L |
| NEU% | 77.1 | 50.0-70.0 | % |
| LYMPH% | 16.0 | 20.0-40.0 | % |
| MON% | 6.3 | 3.0-10.0 | % |
| EOS% | 0.5 | 0.5-5.5 | % |
| BAS% | 0.1 | 0.0-1.0 | % |
| NEU# | 9.11 | 2.00-7.00 | *10**^9^**/L |
| LYM# | 1.89 | 0.80-4.00 | *10**^9^**/L |
| MON# | 0.74 | 0.12-1.00 | *10**^9^**/L |
| EOS% | 0.06 | 0.05-0.50 | *10**^9^**/L |
| BAS% | 0.01 | 0.00-0.10 | *10**^9^**/L |
| HCT | 39.6 | 42.0-49.0 | % |
| MCV | 81.5 | 82.0-95.0 | f1 |
| MCH | 29.0 | 27.0-33.0 | pg |
| MCHC | 356 | 320-360 | g/L |
| RBC-CV | 11.9 | 11.0-15.0 | % |
| RBC-SD | 35.1 | 39.0-46.0 | f1 |
| PCT | 0.280 | 0.108-0.282 | % |
| PDW | 8.8 | 9.0-17.0 | f1 |
| MPV | 8.7 | 7.8-12.5 | f1 |
| P-LCR | 13.70 | 13.0-43.0 | % |
| CRP | 10.26 | <8.00 | mg/L |
| TP | 66.0 | 63-82 | g/L |
| ALB | 42.0 | 35-50 | g/L |
| GLO | 24.0 | 20-40 | g/L |
| ALT | 24.0 | 5-69 | U/L |
| AST | 33.0 | 15-46 | U/L |
| ALKP | 165.0 | 38-126 | U/L |
| GGT | 13 | 12-58 | U/L |
| LDH | 177 | 94-250 | U/L |
| TBIL | 9.4 | 3.0-22.0 | umol/L |
| UREA | 5.0 | 2.5-7.1 | umol/L |
| CREA | 41.0 | 62-133 | umol/L |
| URIC | 265.0 | 140-414 | umol/L |
| GLU | 4.5 | 3.6-5.8 | mmol |
| Ca | 2.4 | 2.10-2.55 | mmol |
| K | 4.0 | 3.5-5.3 | mmol |
| Na | 138.0 | 135-150 | mmol |
| CL | 102.0 | 96-108 | mmol |
| CO2CP | 24.0 | 21-31 | mmol |
| AG | 16.0 | 4.00-20.00 | mmol |
| PT | 11.4 | 9.5-15.0 | sec |
| PT-INR | 1.04 | 0.80-1.50 |  |
| APTT | 31.8 | 20.0-40.0 | sec |
| TT | 17.3 | 13.0-25.0 | sec |
| FIB | 3.24 | 1.80-4.00 | g/L |
| D-Dimer | 177.0 | 0-500 | ng/ml（FEU） |
| GLU | （-） | （-） |  |
| NIT | （-） | （-） |  |
| KET | （-） | （-） |  |
| SG | 1.018 | 1.005-1.030 |  |
| BLD | （-） | （-） |  |
| PH | 6.50 | 5.50-8.00 |  |
| PRO | （-） | （-） |  |
| UBG | normal |  |  |
| BIL | （-） | （-） |  |
| WBC | （-） | （-） |  |
| VC | （-） | 0.0-0.5 | mmol/L |
| RBC | 0 | 0-0 | /HP |
| WBC | 0 | 0-0 | /HP |

The measurement was performed on November 7th, 2020 before the surgery day. WBC#: white blood cell count; RBC#: red blood cell count; PLT#: platelet count; NEU%: neutrophil percentage; LYMPH%: lymphocyte percentage; NEU#: neutrophil count; HCT: hematocrit; MCV: mean cell volume; RBC-SD: red blood cell distribution width-standard deviation; PDW: platelet distribution width; CRP:C-reactive protein; ALKP: alkaline phosphatase; CREA: creatinine.

Hematoxylin–Eosin Staining


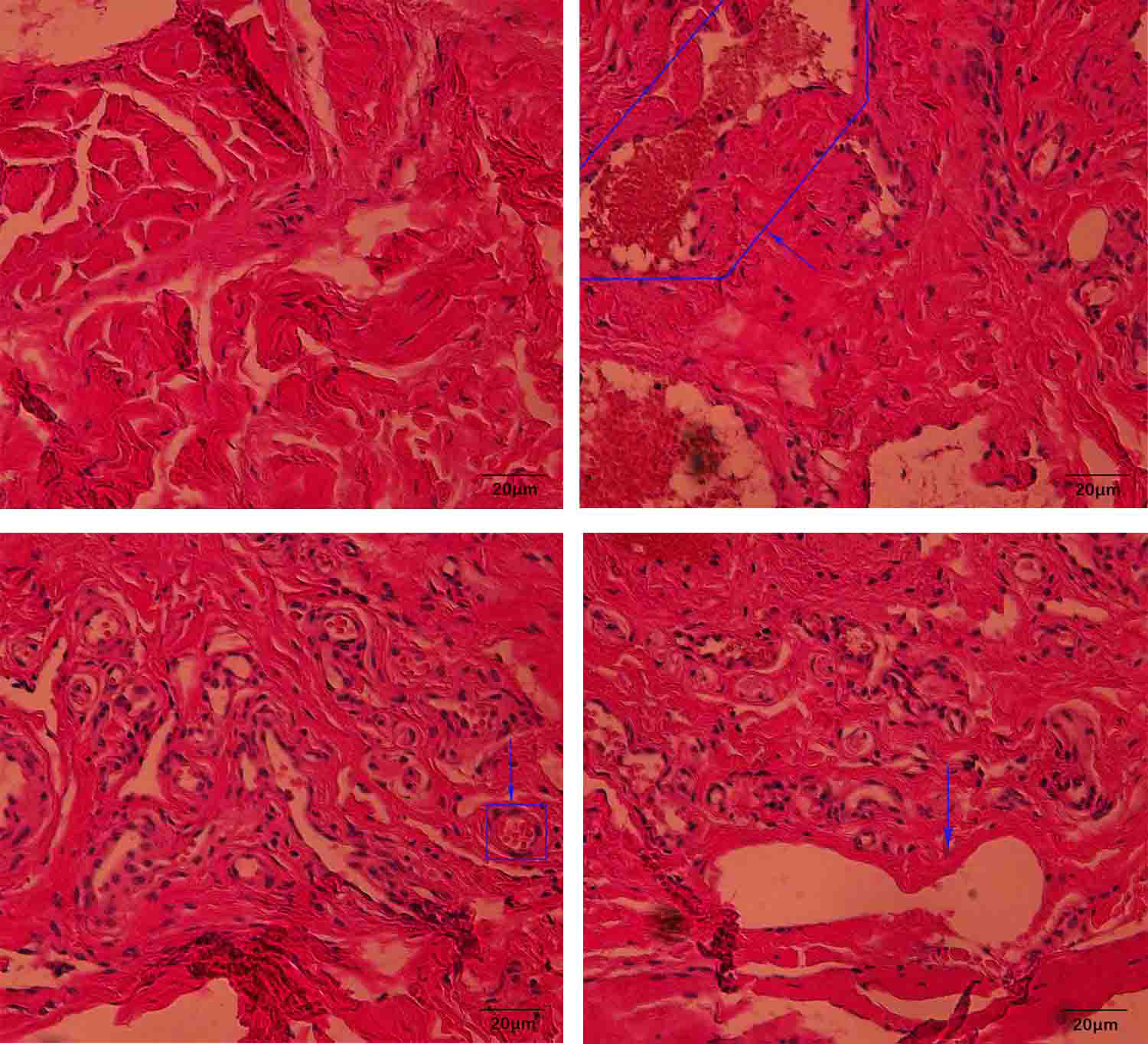


**Figure S3. Hematoxylin–Eosin (HE) Staining.** Connective tissue (top-left subgraph) are most abundant in the tumor sample, A dilated capillary (top-right subgraph), a normal capillary (bottom-left subgraph) and a lymphatic vessel (bottom-right subgraph) are indicated by blue arrows.

After the surgery day November 11^th^, 2020, sections were dipped into gradient ethanol, stained with hematoxylin for 10 minutes, differentiated in 1% (v/v) hydrochloric acid alcohol for 5–10 seconds, and stained with eosin for 10 minutes. The sections were rinsed with distilled water for each interval step, and then dehydrated with gradient ethanol, cleared with xylene, mounted with neutral gum, dried, and observed using light microscopy (**Figure S3**).

Immunohistochemistry experiments

Among the 63 coding genes in the gene-expression signature, *UCHL1* was identified as the best one for discriminating embryonic MSCs from other cells. Then, we applied immunohistochemistry (IHC) technique to detect MSCs by the antibody of UCHL1. As a result, stained cells in the tumour tissues of three patients are embryonic MSCs, which are characterized by three features (**Figure 3**)：(1) the cytoplasm, not the nuclei (in oval shape) is stained by IHC; (2) the ratio of nuclear size to cell size is slightly smaller than that of an embryonic stem cell; and (3) the cells are often located in the connective tissue. Three patients (Admission number: 1097108, 1089641 and 1082186) diagnosed with angiolipoma, unclassified hemangioma and cavernous hemangioma, are 1 years old girl, 5 years old girl and 14 years old boy. *UCHL1* can be used as a marker gene to detect embryonic MSCs at different apoptosis stages.

Paraffin-embedded tumor samples were sectioned at 4 microns. The sections were dewaxed in xylene and ethanol, then subsequently submerged in 10 mM citrate buffer (pH 6.0) at 95 °C for 20 min in a microwave oven. Followed by washing in phosphate-buffered saline (PBS) for 3 times, and endogenous peroxidase was halted by the addition of 3% H_2_O_2_, and then incubated at room temperature for 15 min. After blocking of endogenous peroxidase and the sections were incubated overnight at 4°C with anti-UCHL1 antibody (OriGene, CHINA) and incubated with the secondary antibodies at room temperature for 30 min. Chromogenic reactions were carried out according to the DAB Kit. The slides then were lightly counterstained with hematoxylin, dehydrated with ethanol, cleaned with xylene, and mounted.
